# Bean Cuisine: The Potential of Citizen Science to Help Motivate Changes in Pulse Knowledge and Consumption

**DOI:** 10.3390/foods12142667

**Published:** 2023-07-11

**Authors:** Chelsea Didinger, Marisa Bunning, Henry J. Thompson

**Affiliations:** 1 Department of Food Science and Human Nutrition, Colorado State University, Fort Collins, CO 80523, USA; chelsea.didinger@colostate.edu (C.D.); marisa.bunning@colostate.edu (M.B.); 2 Cancer Prevention Laboratory, Colorado State University, Fort Collins, CO 80523, USA

**Keywords:** beans, pulses, legumes, citizen science, public health, Extension, community engagement

## Abstract

Pulses, or the dry, edible seeds of non-oilseed legumes (e.g., chickpeas, cowpeas, dry beans, dry peas, and lentils), are uniquely positioned to simultaneously benefit human and environmental well-being, all while being affordable and important to diverse cultural food traditions around the world. Despite the benefits they can provide, pulses are dramatically under-consumed. One key barrier preventing higher intake among consumers is a lack of familiarity with how to prepare and regularly incorporate pulses into meals. To address this barrier and actualize findings from our laboratory, we created the Bean Cuisine, a 2-week cuisine (i.e., meal plan) with 56 pulse-centric recipes corresponding to 14 unique breakfast, lunch, snack, and dinner ideas. Each meal category was largely interchangeable, i.e., the order of the breakfast recipes is not important, and one could be swapped for another if a different order were preferrable to a consumer. Fifty-six citizen scientists were recruited to provide feedback on the Bean Cuisine. Free response feedback related to project participation was very positive, and common themes included changes in pulse consumption and cooking behaviors, increased awareness of pulse variety and versatility, and positive perceptions of citizen science. Overall, participation in the Bean Cuisine citizen science project helped create pulse advocates, empowering participants to advance the well-being of their communities through pulses.

## 1. Introduction

Pulses are a nutrient-dense food that can be a staple in healthy dietary patterns supportive of public and planetary health. Health benefits associated with pulse consumption range from improving gut health and healthy weight management to the prevention of chronic diseases such as type 2 diabetes, cardiovascular disease, and cancers like colorectal cancer [1]. Individuals who regularly consume pulses have been found to have more nutrient-dense diets, including higher intakes of fiber, folate, and potassium and lower consumption of fat [2]. Although more research is needed to improve the quality of the evidence and build more robust support for the exact health benefits associated with pulses—as well as the amounts and types of pulses that support optimum health—this demonstrates the potential of pulses to improve diet quality. The environmental contributions of pulses include benefits for soil health, lower water requirements in comparison to other crops and sources of protein, and the potential to reduce greenhouse gas emissions [3,4,5].

In addition to their human and environmental health benefits, pulses have been grown around the world for millennia [6] and therefore are a traditional part of diverse cuisines [7]. Moreover, pulses are known for being available at an economical price point, one of the reasons that the Food and Agriculture Organization of the United Nations emphasizes the ability of pulses to advance food security [7].

Yet, pulses are under-consumed, which prevents us from capitalizing on their many benefits. In the United States, most people eat less than 1 cup of cooked pulses per week [8]. Globally, pulse intake is also shockingly low, estimated at around only 21 g per day, although, of course, this can vary by country and population [9]. For example, per capita, bean consumption in Eastern Africa is significantly higher, with people annually eating their body weight (50–60 kg per year, or about 137–164 g per day) in Rwanda, Burundi, and eastern Kenya [10]. Shifting the eating behavior curve so that the global population has a higher level of pulse intake can result in a win-win situation for the health of people and the planet.

There are several key barriers to higher pulse consumption, including long cooking times and concerns about digestion and flatulence [11]. Among these barriers, a primary reason for the low consumption of pulses is consumer unfamiliarity with how to prepare pulses and regularly include them in their daily diets by taking advantage of the culinary versatility of pulses [11,12,13]. One way to highlight the diverse applications of pulses is by providing the public with accessible, tasty, and creative recipes. However, the mere creation of recipes does not guarantee that people will necessarily try them. Also, the public may not have the background knowledge about the numerous benefits of pulses, nor tips on how to prepare them, to ensure they have positive experiences that will encourage them to continue cooking with pulses. Actively engaging the public through citizen science efforts is one approach to mitigating barriers to the regular inclusion of pulses in the diet, with the important added benefit of collective learning wherein the public can actively participate in research while scientists also learn from the citizen scientists. Through the Bean Cuisine project, the research team went one step further, providing not only recipes and taking steps to ensure positive experiences but actually creating a dietary pattern and menu defined by the recipes, focusing on pulses as a staple food.

Vohland and colleagues state that “citizen science broadly refers to the active engagement of the general public in scientific research tasks” [14] (p. 1). Citizen science is becoming increasingly popular, with millions of people contributing to data collection for a wide variety of projects annually [15]. Multiple benefits to science and the participants themselves are recognized, such as increasing scientific knowledge, providing educational opportunities for citizen scientists and the chance to engage in the research process, helping to democratize data collection, and offering the potential to involve a larger audience than traditional research methods and capitalize upon the diversity of ideas generated by larger numbers of people [15,16,17]. Citizen science has been used in an array of disciplines, but it is still relatively lacking in nutrition and food research, which can have a great impact on public health [16,18,19]. Citizen science was a practical approach for this research project because it resulted in feedback on the home cooking experience, reflective of how people will actually prepare and perceive the recipes that are part of the Bean Cuisine. Although it included a variety of pulses (i.e., the dry, edible seeds of non-oilseed legumes, including chickpeas, cowpeas, dry peas, and lentils) in addition to beans, it was called “Bean Cuisine” instead of “Pulse Cuisine” due to higher familiarity with the word bean and the bean-centric nature of the cuisine. However, it is also attractive to a broader population due to its inclusion of multiple pulse types. Due to the name of the cuisine being Bean Cuisine, the words ‘pulse’ and ‘bean’ may appear to be used largely interchangeably in this paper.

The Bean Cuisine was created to address the barrier of unfamiliarity with pulses, instead inspiring citizen scientists with the culinary versatility of beans and other pulses, encouraging regular inclusion in their daily diets. This represents a type of stealth health approach wherein participation in the project provides the opportunity to begin to routinize regular pulse consumption. Additionally, the Bean Cuisine presented an opportunity to share with the public recent preclinical findings, which suggest that major benefits for gut health and body weight management are achieved when at least 35% of dietary protein comes from pulses [20,21]. The Bean Cuisine thus also helps visualize what a diet that attains this level of pulse consumption may look like and the feasibility of eating this level of pulses. By using the feedback provided by citizen scientists, the Bean Cuisine was improved so that it will be more accessible and palatable to a wider audience. In addition, the research team had several key takeaways to improve future citizen science work.

## 2. Materials and Methods

### 2.1. Creation of the Bean Cuisine

The Bean Cuisine was created to provide 35% of total dietary protein from pulses. To spread consumption throughout the day and address the consumer barrier of unfamiliarity with how to use pulses, 14 days of recipes that included pulses for breakfast, lunch, snack, and dinner were created, 56 recipes in total. Recipes were collected from colleagues, including Dr. Terry Hartman, who provided bean recipes she had previously used in a clinical trial [22]. Additionally, pulse-centric food blogs and cookbooks were searched to gather recipes. Recipes were then modified to ensure that 35% of dietary protein each day came from pulses and that the cuisine also met other recommended intake levels of macro- and micronutrients. Specifically, the Bean Cuisine had recipes that included black-eyed peas, chickpeas (in flour and whole form), split peas, lentils (in whole and pasta form), and many types of dry beans, including black, Mayocoba, pinto, red kidney, and assorted white beans (e.g., great northern, cannellini, navy), and it did not include use of green, immature pulse grains.

The recipes emphasized the versatility of pulses, including applications such as:

- Sweet and savory baking with pulses (mashed cooked pulses and chickpea flour);
- Pulse pasta (e.g., lentil pasta) to demonstrate simple substitutions for ingredients normally used (e.g., wheat pasta);
- Combining beans with other grains, such as brown rice, quinoa, or oatmeal;
- Combining beans and meat;
- Simple, quick recipes like savory and sweet bean dips;
- A range of easy-to-prepare pulse salads.

The recipes were assembled into a 2-week menu plan in Nutritionist Pro (version 8.1.0, 2022, Nutritionist Pro, Axxya Systems). Citizen scientists were only provided with pulse-based recipes, but to confirm that 35% of participants’ overall daily protein was coming from pulses, sides (e.g., fruit, yogurt, and beverages) were added to Bean Cuisine in Nutritionist Pro as well. The average daily intake of pulses was about 2.5 cups of cooked pulses, which totaled about 38 g of protein from pulses.

An analysis to estimate energy, macro-, and micronutrient intakes was conducted on pulse recipes, daily intake, and the overall Bean Cuisine (see Supplementary Materials S1 for nutrient analysis and Supplementary Materials S2 for the meals report). Overall, the Bean Cuisine proved to be a healthy diet that met dietary guidelines (Nutritionist Pro is occasionally missing micronutrient levels for ingredients, which is why the percentages of a select few micronutrients—like biotin at 71%—may appear slightly low). The Bean Cuisine with added sides resulted in average daily intakes of approximately: 1999 kilocalories, 292 g of carbohydrate, 59 g of fat, and 96 g of protein. This is roughly 39.8% of total protein intake from pulses, based on numbers provided by Nutritionist Pro. Importantly, 2 key dietary components of public health concern in the United States, dietary fiber and potassium [8], were consumed in adequate amounts. Total dietary fiber intake was around 61 g, with 5143 mg of potassium. Also, cholesterol, saturated fat, and added sugars were present in low amounts in Bean Cuisine.

### 2.2. Citizen Scientist Recruitment and Assignment

Colorado State University Extension and colleagues helped recruit a convenience sample of citizen scientists through emails, newsletters, and social media posts. Fifty-eight citizen scientists filled out the intake form created in Qualtrics. Each individual was then contacted via email to confirm participation. As email would be the main form of communication, we wanted to ensure they would respond. Fifty-six of the 58 participants responded, resulting in a total of 56 citizen scientists recruited to the Bean Cuisine project. There were 14 days in the project, and each individual was assigned 1 day (i.e., a breakfast, lunch, snack, and dinner recipe), resulting in 4 citizen scientists being assigned to each day of the Bean Cuisine.

The intake form asked about the participants’ understanding of the commitments relating to participating in the Bean Cuisine project, motivation for participating, preparation and consumption habits for pulses, knowledge about pulse-related topics, and demographics. See Supplementary Materials S3 for the intake form. In addition, the intake form included questions about participants’ food allergies, intolerances, and preferences. Assignment to days was made based on citizen scientists’ dietary patterns and preferences. After those with dietary restrictions had been assigned, the remaining participants were randomly assigned to the open spots.

**Figure 1:**
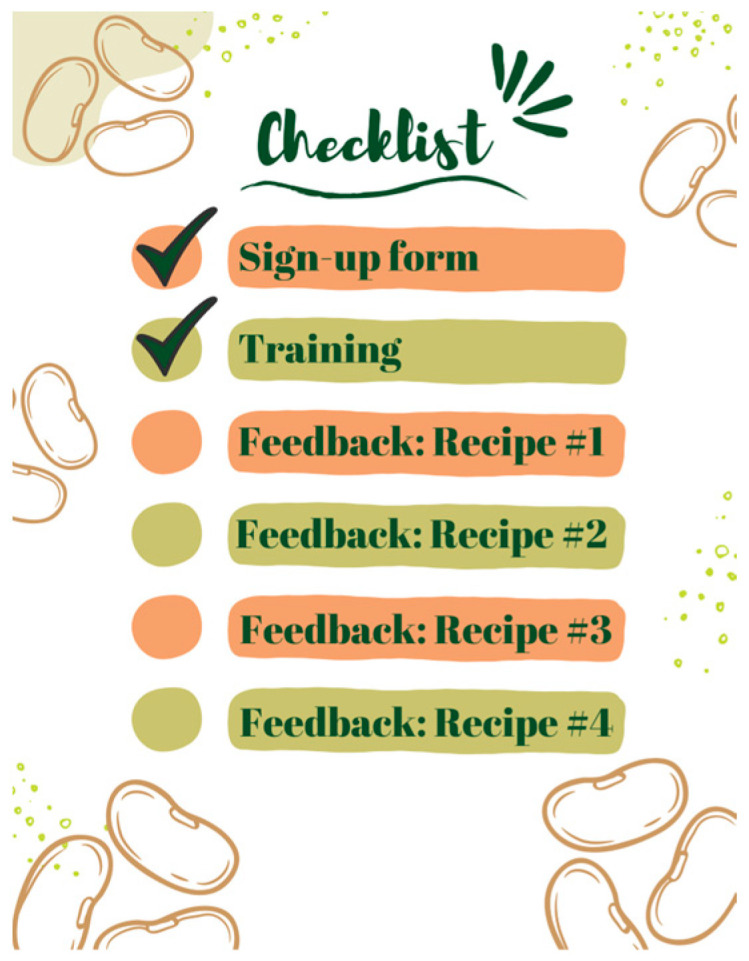
Citizen scientist checklist. This is the graphic that was shown to citizen scientists in the online training session detailing participation in the Bean Cuisine project.

Participation as a citizen scientist included 6 main components (see Figure 1): the intake form (also called the sign-up form), training, and then the completion of 4 feedback forms corresponding to the 4 recipes each individual was assigned. This project was approved by the Colorado State University Institutional Review Board, protocol #3589.

### 2.3. Citizen Scientist Kit and Training

The training served as a chance for citizen scientists and the research team to interact with one another. It also helped ensure that citizen scientists clearly understood their role in the project by providing details and the chance to ask clarifying questions. Training is also a way to improve data quality in citizen science [23,24]. Replication of data across volunteers is another way to have better data quality [24], hence the recruitment of 4 volunteers for each recipe.

**Figure 2:**
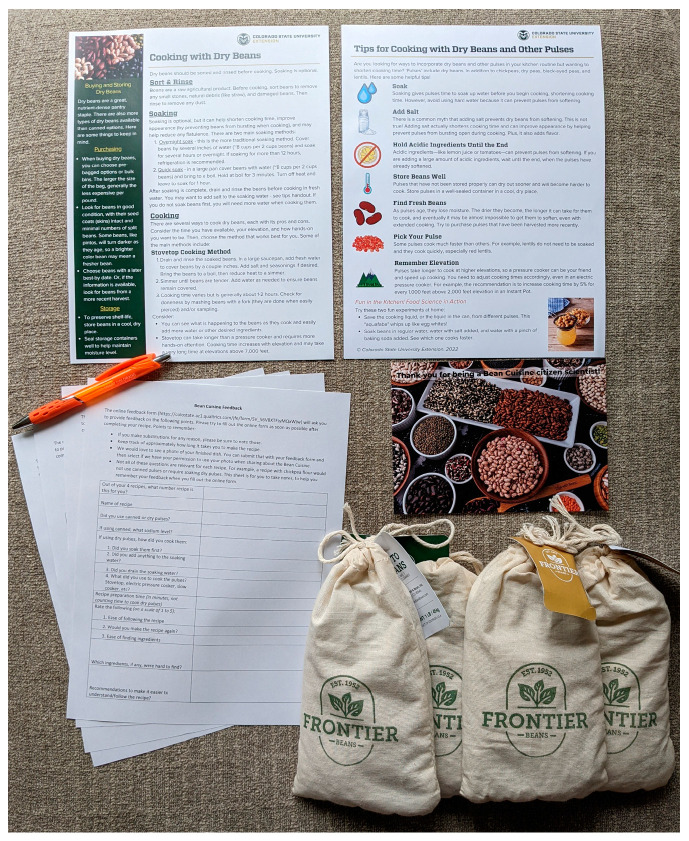
Citizen scientist kit. An image of contents in the kit mailed to citizen scientists.

Before the training, citizen scientists were all sent a citizen scientist kit (see Figure 2). The kit included:

- Two CSU Extension handouts were created as part of this project: 1 on cooking with dry beans and the other on tips to shorten cooking time;
- A recipe packet that included 1 explanation page with a QR code that linked to the recipe feedback form, as well as printed copies of all 4 of the recipes they had been assigned;
- A printed form detailing what they could expect to be asked about in the online feedback form—all feedback needed to be submitted virtually, but the form was provided so people could take notes on it if desired;
- A thank you;
- A pen;
- Four 1-lb bags of Colorado Proud* beans: 2 bags of pinto beans and 2 bags of Mayocoba beans (*for this Colorado Department of Agriculture funded project, all beans provided to citizen scientists were grown in Colorado or a neighboring state and processed in Colorado).

The citizen scientist kits arrived around the scheduled day of the training, and participants were told to wait to begin until completing the training. Before beginning recipe feedback, citizen scientists participated in the training in 1 of 2 ways:

1. **Live training**. We provided a 1 h online training session via Zoom videoconferencing on a weekday evening to make participation easier. The research team briefed citizen scientists on the project background, explained the details of participation, and then held a Q&A. Roughly 60% of citizen scientists joined the live training. Participants were highly engaged, asking questions in the chat and unmuting to ask live questions to clarify details about expectations for project participation. For instance, participants asked about ingredient substitution and the ideal timeframe within which to complete all recipe feedback. Often, answers were already available in the instructions they received in the citizen science kit (e.g., sticking to the recipes as close as possible given dietary restrictions and preferences and ingredient availability, and noting any substitutions made in the feedback form). Additionally, some questions pertained to work being conducted at Colorado State University to learn more about current research on the health benefits of pulses that the research team is conducting (e.g., gut health promotion and chronic disease prevention);
2. **Recorded training**. We created an approximately 22 min recorded training session that performed a similar function but did not include the live Q&A for those who were unable to join the live training. Citizen scientists were encouraged to reach out with any questions. The live training can be found at the following link, which was posted as a private video (to share with citizen scientists, not for general viewing by anyone on YouTube), available at https://youtu.be/QYtLk-piuW0.

Immediately upon completion of the live training, an email was sent to all citizen scientists thanking them and including attachments of all the printed forms they had received printed in their kit (2 handouts, a recipe packet, and a PDF on what details to expect in the feedback form), for those who prefer digital copies. A link was also provided to the recorded training for those who could not join live or wanted to watch the training again. Lastly, a direct link to the feedback form was also provided. The first time that citizen scientists completed the feedback form, they needed to first confirm they had completed the training.

### 2.4. Citizen Scientist Feedback Form

To simplify things and reduce any potential confusion, only 1 link and associated QR code were provided to access the feedback form created in Qualtrics. Skip logic was implemented so that one of the first questions inquired, “Of the 4 recipes about which you will provide feedback, what recipe number is this for you?” For the first feedback form, participants had to confirm completion of the training and were asked to provide feedback on the training. The second and third time that participants completed the form, there were no additional questions beyond the general recipe feedback. General feedback asked for details such as:

- Whether they used canned or dry pulses they cooked themselves;
- Details about the canned pulses (e.g., sodium level) or how they prepared the dry pulses (e.g., soaking, how they cooked them—stovetop, pressure cooker, slow cooker, etc.);
- The approximate time it took to prepare the recipe;
- Level of agreement with the recipe being easy to understand, inclination to make the recipe again, and ease of procuring the ingredients;
- Recommendations about improving recipe instructions;
- Which ingredient(s) were hard to find, if any;
- Satisfaction with taste, texture, appearance, and overall acceptability of the recipe (i.e., sensory feedback);
- Whether they followed the recipe or made substitutions, and if so, details of the substitutions;
- What pulses they may recommend using in place of those already recommended in the recipe;
- What changes they would recommend making to the recipe;
- Submit a photo of the finished dish, if so inclined.

The fourth and final time they completed the form, they were asked about their pulse preparation and consumption habits, knowledge level, and overall experience participating in the project. See Supplementary Materials S4 for the feedback form. After completing their final feedback form, citizen scientists were sent a $20 Amazon gift card as a thank you for their valuable feedback and participation in the project.

### 2.5. Statistical Analyses

All statistical analyses were conducted in IBM SPSS Statistics version 28 (SPSS Inc., Chicago, IL, USA). Frequencies and percentages were calculated for categorical variables like demographics. Descriptive statistics were used on categorical variables like demographics. For Likert-scale questions about knowledge and behavior that were asked of citizen scientists when they enrolled and again upon completing the project, Wilcoxon signed-rank test was used to assess if significant changes occurred. For free response questions, the lead author took notes and developed a set of summary comments, which were then grouped into themes. The themes were corroborated by the other researchers on the project.

### 2.6. Modifying the Bean Cuisine

The response rate was 100%, with all 56 citizen scientists completing feedback forms for each of their 4 assigned recipes. The recipes were ranked by computing an average score for each of the 7 Likert scale questions that asked about recipe preparation and sensory evaluation (1. ease of following and understanding the recipe, 2. ease of finding the ingredients, 3. inclination to make the recipe again, and satisfaction level with 4. taste, 5. texture, 6. appearance, and 7. overall acceptability). Then, the average scores were totaled to assign an overall score to each recipe. The most important questions were deemed to be agreement with the statement “I would make this recipe again” and satisfaction with “overall acceptability,” as these reflect the chance people will continue to use the recipes and provide an idea of overall sensory appeal. Citizen scientists responded using 5-point Likert scales, where 1 or 2 reflected a positive score, 3 was neutral, and 4 or 5 represented dissatisfaction. Accordingly, recipes that received a 3 or higher for one of these questions were prioritized for modification and further recipe testing, as participants were only neutral or potentially disliked the recipe. Data shown in sensory tables has been reverse coded so that 5 is a high score and 1 a low score to be consistent with the scaling of the knowledge tables.

In addition, all recipes were modified based on citizen scientist feedback to free response questions. This included improvements to clarify recipe instructions and adjustments to ingredient amounts. For sample feedback, see the results section on citizen scientist feedback. Citizen scientists were also asked how long it took to prepare a recipe, and the times were averaged to provide a time estimate for the final versions of the recipes. Any modifications to ingredients that were made were also entered into Nutritionist Pro, and the resulting nutrition facts were included in the final recipe. The research team all participated in proofreading and editing the final Bean Cuisine.

### 2.7. Sensory Panel

Validation with experts can improve data quality [24]. Therefore, 4 recipes that had already been modified per citizen scientist feedback were tested using a sensory panel as a way of validating citizen scientist feedback and confirming palatability when the dishes were prepared by the lead author in a university teaching kitchen instead of in the home kitchen. The sensory panel consisted of 12 faculty, staff, and students with experience providing sensory evaluation input, most of whom are in the Department of Food Science and Human Nutrition at Colorado State University. More details about the recipes are provided in the results section about the sensory panel including why this representative subset was selected. The recipes showcased versatile preparations of beans, and panelists were asked to rank 4 components: taste, texture, appearance, and overall acceptability of each dish. A Likert scale from 1–5 was used (1 = extremely satisfied, 2 = somewhat satisfied, 3 = neither satisfied nor dissatisfied, 4 = somewhat dissatisfied, 5 = extremely dissatisfied). Panelists also provided written feedback and suggestions for these components, such that feedback could be incorporated into the final Bean Cuisine. Data shown in sensory tables have been reverse coded so that 5 is a high score and 1 a low score to be consistent with the scaling of the knowledge tables.

## 3. Results

### 3.1. Citizen Scientist Demographics

Participants were asked to report their dietary patterns. Most citizen scientists were omnivores (*n* = 39), followed by vegetarians (*n* = 8), pescatarians (*n* = 5), and vegans (*n* = 4). About 96% of citizen scientists were female, and the two most reported age groups were 60–69 (*n* = 19) and 50–59 (*n* = 15). The 40–49 and 70–79 age groups each had *n* = 7 participants. Fifty-three participants were Caucasian, and most citizen scientists had either a Bachelor’s (*n* = 24) or Master’s (*n* = 22) degree. Most participants were from Colorado (*n* = 44), as this was a Colorado-based project. However, volunteers from a total of 10 states participated. A table showing more detailed descriptive statistics about participant demographics can be found in Supplementary Materials S5.

### 3.2. Citizen Scientist Feedback and Bean Cuisine Modification

As explained above, part of the recipe feedback included responding to seven 5-point Likert scale questions. The average score for all 56 recipes is shown in Table 1.

All recipes were modified based on citizen scientist feedback. For recipes that scored well and received positive feedback, modifications were minor, such as improving wording clarity, proposing optional additions to boost flavor, or incorporating tips from citizen scientists. Recipes that received lower scores (as previously detailed) were tested again by the research team. Common modifications included:

- Specifying that the amount of pulses listed is the cooked amount (example feedback: “Write whether the bean amount is for cooked or uncooked beans for clarity.”);
- Providing clarity on how to cook dry lentils or split peas for recipes that included them (example feedback: “I had to look up how to cook red lentils. It would have been nice to have those with the recipe.”);
- Increasing seasoning level (example feedback: “Add seasonings, possibly cumin, chili powder, taco seasoning and/or salt.”);
- Adding more details to the instructions (example feedback: “Add instructions like ‘do not overmix’ [and] ‘bake in the middle rack of the oven.’”);
- Clarifying time estimates in intermediate steps (example feedback: “It would have been better to have a range like 15–20 min or until tender.”);
- Suggesting serving options (e.g., with rice) and toppings (example feedback: “Pairing it with a carbohydrate like rice. Recommending avocado as a topping.”).

On the recipe feedback forms, citizen scientists were asked if they followed the recipe. Despite being assigned recipes that avoided food allergies and dietary restrictions and receiving instructions to please follow the recipe as closely as possible, people only indicated following the recipe 54% of the time.

### 3.3. Impacts of Participation

Citizen scientists were asked about their knowledge level on three different topics prior to participation and upon completion of the project. They were also asked about preparation and consumption behaviors. The data is shown in Table 2.

**Figure 3:**
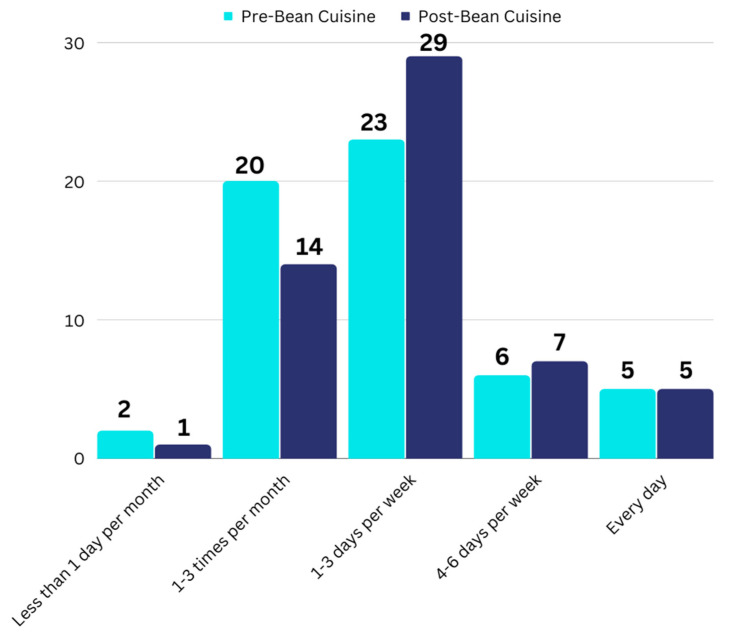
Changes in frequency of pulse consumption. “Less than 1 day per month” is shortened in the figure, but the full response option read “Several days per year, but less than 1 day per month”. No participants selected “Never” as the response for how often they eat pulses. The actual numbers of responses out of *n* = 56 are shown above each column.

Changes in the frequency of pulse consumption were also seen, as shown in Figure 3. The most noticeable shift is that more participants reported eating pulses 1–3 days per week than before the project. However, although the overall frequency of consumption appears to be increasing, the overall change was not found to be significant (*p* = 0.137), a point further expanded upon in the discussion.

**Figure 4:**
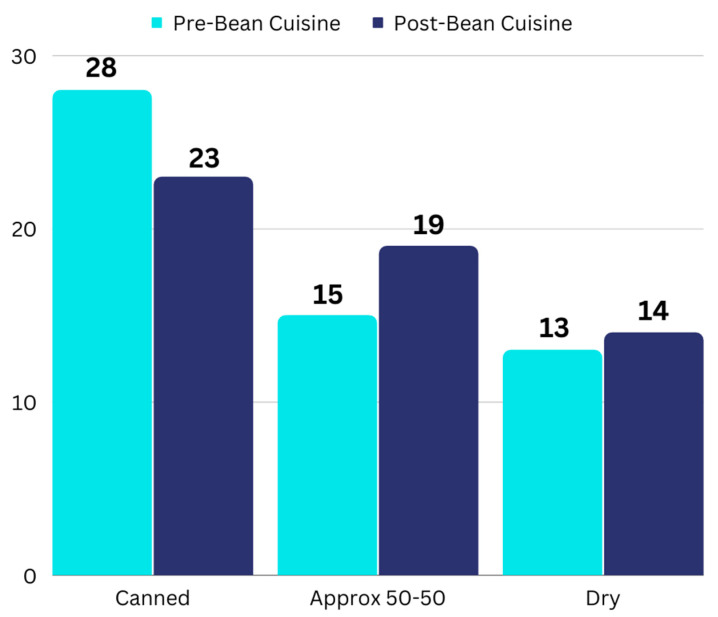
Citizen scientist usage of dry versus canned pulses, pre- and post-participation. “Approx 50-50” indicates that the participants used canned pulses and pulses cooked in the home with similar frequency. The numbers of responses are shown above the column.

Citizen scientists also indicated their frequency of usage of canned pulses versus dry pulses they cooked at home. Results are reported in Figure 4, which shows an overall trend of increasing use of dry pulses after participation. This is most apparent in changes observed with several individuals who primarily used canned pulses before the project but later reported a similar frequency of use of canned and dry cooked in the home, indicative of more frequent cooking of dry pulses. The greater frequency of cooking dry pulses in the home is reflected in the free responses as well.

As this project was funded by a Colorado Department of Agriculture grant, participants were also asked upon enrollment whether they regularly purchased Colorado-grown beans. The most common response to this question (*n* = 25, 44.6%) was “unsure if the beans I purchase are from Colorado or not.” Participation in the project resulted in 39 individuals (69.6%) indicating they are now more likely to purchase Colorado-grown pulses.

### 3.4. Participation in Citizen Science

Recurring themes and examples of quotes pertaining to these themes are shown for citizen scientists’ motivations for participation (Table 3) and their overall experience (Table 4).

The evaluation of overall experience (Table 4) is derived from quotes provided during individual recipe feedback in addition to the final feedback form when citizen scientists were explicitly asked about their experience in the project. Common participation outcomes in citizen science projects are grouped into categories such as interest, motivation, knowledge, behavior, attitudes, skills, self-efficacy, and other personal outcomes [25,26]. Free responses from the Bean Cuisine project participants fell into similar categories.

### 3.5. Sensory Panel

Sensory panelists ranked the taste, texture, appearance, and overall acceptability of each dish on a Likert scale of 1 to 5, with 5 being extremely satisfied and 1 extremely dissatisfied. The average scores for all dishes ranked between extremely and somewhat satisfied. Table 5 shows the reason a dish was included in the sensory panel, quotes about the dish from sensory panelists, and the average overall acceptability score (*n* = 12). Similar to citizen scientists, perception of the recipes differed, and the very same recipe received a range of scores, from rankings of ‘extremely satisfied’ to dissatisfied. As shown in the last column of Table 5, the range of scores provided varied more for the dip and skillet than the smoothie and salad, with the one panelist who did not like the skillet reporting it tasted “very sweet”, which the individual found unappealing.

### 3.6. Final Bean Cuisine

Overall, the final version of the Bean Cuisine resulted in similar levels of total pulse intake as the original version. The original version contained an average of about 2.5 cups (406.6 g) of cooked pulses per day, and the final modified version contained 2.4 cups (388.8 g). Cooked weights provided by Nutritionist Pro were used for these calculations. To calculate the cooked weight of dry pulse ingredients, the dry weight of chickpea flour, lentil pasta, and dry lentils was multiplied by 2 to reflect water uptake. For the final Bean Cuisine PDF that was provided to citizen scientists, optional sides were not included so that citizen scientists could easily use and adapt the recipes to their dietary patterns and share them with friends and family to inspire others with the versatility of pulses. Moreover, as previously mentioned, each meal category was largely interchangeable, so the order in which the breakfasts, lunches, snacks, and dinners are eaten is not important.

Supplementary Materials S1 shows the nutrient analysis provided by Nutritionist Pro of the original 14-day Bean Cuisine, which includes all 56 pulse-centric meals (i.e., 14 breakfasts, lunches, snacks, and dinners), plus sides (e.g., beverages, yogurt, fruit, grains, and vegetables). The Original Bean Cuisine provides roughly 2000 kilocalories, 96 g of protein (~38 g protein from pulse, or ~40% of total protein), 61 g of dietary fiber, 5143 mg of potassium, and 1934 mg of sodium per day. Micronutrient levels are not available for all the ingredients in the Nutritionist Pro system, hence why some percentages of micronutrients may appear low.

Supplementary Materials S6 shows the nutrient analysis for the Updated Bean Cuisine, with only the pulse recipes updated and the original sides left as they were. The Updated Bean Cuisine provides approximately 2037 kilocalories, 98 g of protein (~37 g protein from pulse, or ~38% of total protein), 61 g of fiber, 5291 mg of potassium, and 2334 mg of sodium. One of the main adjustments made to the Bean Cuisine was increased seasoning levels, as is evident in the increased sodium content. However, exact nutritional content can vary with brand and other factors, and the numbers provided by Nutritionist Pro serve as an estimation. Also, seasoning amounts are optional (i.e., people could use less salt), and sides were not adjusted. If sides were also modified, then the calorie and sodium content could easily match that of the Original Bean Cuisine. However, they were kept the same to reflect changes that occurred strictly due to feedback from citizen scientists.

**Figure 5:**
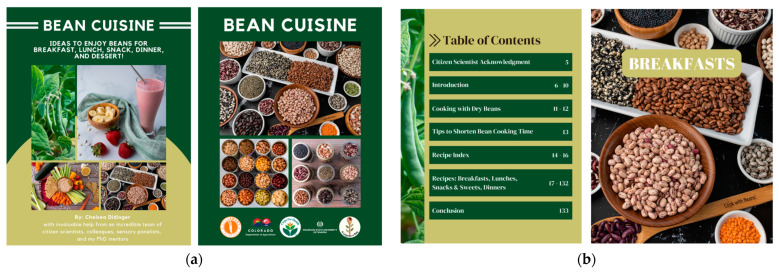
Bean Cuisine. (**a**) Bean Cuisine front and back covers; (**b**) table of contents and Breakfast divider section as an example divider section.

The final version of the Bean Cuisine was a 134-page PDF that was emailed to all 56 citizen scientists, as everyone indicated they wanted to receive the final copy. The front and back covers are shown along with the table of contents in Figure 5. An acknowledgment recognized the critical input provided by the citizen scientists. The introduction included background details about the project and the importance of pulses, as well as helpful handouts on how to prepare dry pulses. Recipes were grouped into four categories—breakfasts, lunches, snacks and sweets, and dinners—with 14 recipes in each for a total of 56 recipes. Each recipe had a title and contained information about the number of servings, a preparation time estimate, a photo of the finished dish, and nutrition facts. Photos provided by the citizen scientists were prioritized, with photos taken by the lead author during recipe testing used as needed. The recipes also listed ingredients and instructions, and they contained a notes section with helpful tips, including tips directly from the free response feedback provided by citizen scientists. The full Bean Cuisine can be found on the laboratory website: https://agsci.colostate.edu/cropsforhealth/.

## 4. Discussion

### 4.1. Bean Cuisine Creation

This creation of the Bean Cuisine serves as a proof-in-concept that 35% total dietary protein provided from pulses is achievable, and the resulting dietary pattern meets dietary guidelines. The preclinical studies demonstrating the potential gut health and healthy weight outcomes of attaining this percentage of protein also examined the 70% protein level. However, when developing the Bean Cuisine, we found that attaining 70% of total protein coming from pulses was very difficult to do while still promoting a varied, healthy diet. This was because most foods contain at least some protein, so including foods like a glass of milk, yogurt, eggs, meat, or even ingredients like whole wheat pasta proved difficult due to the need to maintain 70% of protein coming from pulses. However, it is important to note two things. One, 70% of overall protein coming from plant foods was much more easily attained, and indeed vegan diets demonstrate that all protein can be provided through plant foods. Two, if considering 70% of dietary protein requirements—rather than 70% of total protein—this goal was achievable. In fact, based on estimates by Nutritionist Pro, the updated Bean Cuisine contained approximately 98 g of protein per day, and about 37.5 g of those were from pulses. So, if the protein requirement of 0.8 g/kg body weight is considered [27], this 37.5 g of protein could already attain 70% of the protein requirement for someone who weighs about 65 kg.

Overall, the Bean Cuisine serves as a stealth health approach, wherein healthy eating behaviors are encouraged through participation in the project. Using healthful ingredients to provide healthier—but still tasty—meals is an approach utilized by institutions like the Culinary Institute of America (CIA) as well. For example, the CIA explains that they adopt a stealth health approach to produce healthier recipes and meals, relying on healthful flavoring strategies and techniques instead of sodium, sugar, and saturated fat [28].

All 56 citizen scientists elected to receive the final Bean Cuisine, and several emailed the lead author during the editing process asking when it would be done, indicating they were looking forward to seeing it. Citizen scientists were emailed to ask if they were comfortable having their first name and last initial included on the Acknowledgements page. Fifty-four indicated yes, one declined, and one requested only first name and no last initial. This type of affirmative response suggests that they feel some sense of ownership in the project. Upon receiving the Bean Cuisine, many citizen scientists responded, expressing thanks, positive feedback, and excitement to try the new recipes.

### 4.2. Citizen Scientist Demographics

Higher participation by white, educated females matches the demographics of other citizen science projects [14,29]. A relative lack of sociodemographic diversity is a common limitation faced by many citizen science projects, which may limit generalizability to a larger audience [14,29]. It is of note, however, that although more females participated than males, many citizen scientists engaged their families and friends and incorporated their feedback. For example, on her intake form, one citizen scientist commented that she and her husband would both be participating and that “You’ll be getting us both in the survey responses.” Thus, the demographics of individuals providing feedback likely vary from what is shown in Supplementary Materials S5, and the number of individuals providing feedback exceeds 56.

### 4.3. Citizen Scientist Feedback and Bean Cuisine Modification

Although feedback was positive overall, of course, there was also criticism. One individual claimed to be “underwhelmed” by the recipes, several expressed the desire for more flavor, and another citizen scientist stated, “I would probably be more likely to repeat the recipes if they were a little more flavorful, containing more fat, sweetening agents, and spices.” This reveals a challenge in developing healthy recipes that fall within dietary guidelines, meet a certain level of pulse consumption (35% total dietary protein coming from pulses, in this case), and appeal to everyone. Sensory feedback thus plays a critical role in determining the most and least liked recipes of a target audience and identifying recipes that need improvement [30]. Providing recipes that are largely interchangeable is another way to help ensure that there are desirable choices for people with different taste preferences, i.e., if someone finds one breakfast unappealing, they can switch it with a different breakfast and still attain a similar amount of protein from pulses.

Constructive criticism helped improve recipe clarity and taste. Citizen scientists had different views of overall recipe appeal, with ratings varying even for the same recipe. However, this is also partially due to individual taste preferences and the nature of being assigned recipes. For example, some people were assigned a recipe they would not have chosen for themselves (example quote: “Yuck! I am not a smoothie person. This did not change my attitude towards smoothies.”), which resulted in a low score.

As previously mentioned, citizen scientists only indicated following the recipe 54% of the time. Sometimes modifications were minor, such as increasing the seasoning level. However, other times, modifications entailed multiple ingredient substitutions or completely diverged from the recipe (example: “I am sorry, I went totally off script here, but the result was so good. I hate yogurt, and the dip idea didn’t really fit with our meal plan. So instead, I tossed the chickpeas in the hot sauce/lemon juice/dill/onion powder/parsley, plus 2 tbsp of tahini, and roasted at 375 for 30 min.”). This resulted in feedback that was not always pertinent to the recipe, as significant modifications were made on occasion. Indeed, data quality is a major concern in citizen scientist projects [14,31]. Yet, due to the nature and goal of this project, this type of feedback does not represent low data quality but rather shows increased external validity [31] because it reflects what happens in the home kitchen when people use recipes, as they are prone to modify to suit their tastes. The recipes nonetheless inspired people to try pulses in new ways, thereby expanding their awareness of versatile uses, and it was a more realistic way to test how recipes work in the home kitchen.

Although we ultimately had a 100% response rate, another challenge was receiving feedback on all four recipes from all 56 citizen scientists. Some participants took longer to respond than others and required follow-up emails. Given the nature of a volunteer project, this is understandable. Nonetheless, it presents a difficulty by placing a time burden on the research team to track responses and follow up with participants. Following up with people before beginning the project to reaffirm interest, providing a welcome training kit and gift (Colorado Proud beans), offering training so people understood the project and had the opportunity to engage with the researchers, regularly following up with participants, and providing an incentive upon completion all appeared to be essential in attaining a 100% response rate, and similar approaches can be used in future citizen science efforts.

### 4.4. Impacts of Participation

Baseline knowledge levels of nutrition and health benefits, versatility, and preparation were already relatively high, as shown in Table 2. Other citizen science projects experience this as well, with individuals who are already highly interested in the topic—and thus often more knowledgeable about it—among the main participants [29,32,33]. Nonetheless, knowledge levels did vary, with one citizen scientist commenting upon completion of the project, “It was fun to try new recipes and learn about an ingredient I don’t use all that much.” This suggests that one potential appeal of participating was the chance to simply learn more about food they do not currently use on a regular basis.

As shown in Table 2, citizen scientists’ self-reported knowledge significantly increased for all three categories assessed in this project: nutrition and health benefits, versatile uses of pulses, and how to prepare dry pulses. Knowledge of versatility saw the greatest increase in average score, rising from 3.39 to 4.38, for a total increase of 0.99 on a 5-point Likert scale. This makes sense, given the nature and focus of the project on showcasing different ways to include pulses in every meal. The next greatest average increase was in knowledge of how to prepare dry pulses, which rose from 3.55 to 4.36 (0.81 increase). Citizen scientists were also mailed dry beans and information on how to cook dry pulses, which could have played a role in the increase in knowledge of how to prepare dry pulses. This corresponds with the increased frequency in the use of dry pulses compared to canned, which was seen when comparing preparation habits before and after the project. It is logical that the smallest average knowledge increase was seen for nutrition and health benefits (3.86 to 4.29, for a 0.43 increase) because the only time this topic was really discussed during the project was during the orientation and training. In addition, participants already rated their knowledge of pulse nutrition and health benefits as high before participation (average score of 3.86 out of 5 before beginning).

Although the frequency of pulse consumption appeared to increase (Figure 3), with a greater percentage of individuals reporting eating pulses 1–3 days a week or 4–6 days a week after participation, the result was not found to be significant (*p* = 0.137). This could partly be due to the large percentage of citizen scientists who already regularly ate pulses before this project. Another challenge was the inability to assess detailed pulse intake information due to the nature of the response options; although they asked about the general frequency of consumption, they did not collect data on the exact number of days pulses were consumed and the actual amounts eaten. Indeed, a lack of validated pulse-specific dietary assessments is one of the challenges in advancing pulse research [34].

When asked during the final feedback survey if they now tried to eat more pulses as a result of participating in this citizen scientist project, *n* = 40 (71.4%) of individuals indicated yes. Combined with the overall trend of increased frequency, this suggests that pulse intake increased post-participation. However, it highlights a key takeaway. Arguably, one of the most important outcomes of participation could be increased pulse consumption. The lack of a tool to better assess the types and amounts of pulse consumed thus poses a challenge.

In a recent paper by Henn and colleagues (2022), the authors developed a tool that begins to address some of these concerns [13]. They asked about the frequency of consumption for the different types of pulses in a manner very similar to this study, but in addition to a question about the frequency of overall pulse intake, they also broke it down into pulse type (e.g., a respondent could select that they eat black beans 2–3 times a week). Moreover, they gather information on what situations different pulse types are consumed (e.g., at home, in a restaurant, on the go, at work/school, other), in which form pulses are purchased (e.g., dried, canned, processed products), and how the pulses are eaten (e.g., as a main ingredient, in a side), and what foods are prepared from pulses (e.g., stews, pasta, salad). Gathering such information will be useful in helping better understand consumer behavior and assess changes in frequency and manner of consumption. However, future citizen science efforts should also assess changes to the actual volume of intake, if possible, through a more sensitive tool. Other citizen science work has also found a similar need to develop more sensitive measures to better assess the impacts of participation [35].

Another point of interest was whether participating influenced preparation behavior. Citizen scientists received dry pinto and Mayocoba beans in the mail, as well as handouts with information about how to prepare dry pulses. Figure 4 suggests that post-participation, citizen scientists did seem to use dry pulses more frequently. However, the nature of the response options limited the analysis to descriptive statistics. The most notable change is a shift from using mainly canned options (*n* = 28 before the project) to a more even mix of canned and dry pulses. Participants still showed high usage of canned pulses after completing the project, likely due to the convenience factor [36].

Especially given that not all citizen scientists were from Colorado, it is expected that many of them may not make an effort to purchase Colorado-grown beans. Yet, the fact that the most common response selected on the intake form was “unsure if the beans I purchase are from Colorado or not” (*n* = 25, 44.6%) suggests that bean origin—whether from Colorado or another region—is often unknown and may not play much of a role in purchasing decisions. Participation in the project appeared to increase awareness of origin, with 69.6% of participants indicating upon completion that they are now more likely to purchase Colorado-grown pulses. This highlights the potential of spreading awareness about local products to influence future purchasing decisions in favor of local options. Barriers to local food purchasing include the inability to find identifiably local foods and a lack of time or behavior skills to prepare local foods [37]. This may be the case with dry pulses, as unfamiliarity with how to cook pulses and the versatile ways to use them is an established barrier to consumption [11]. One recommendation by Birch and colleagues (2018) to enhance appeal to consumers was to create clearer branding and labeling [38].

### 4.5. Participation in Citizen Science

The motivations of participants to join this project (see Table 3) match those in other citizen science projects, which include intrinsic interest in the project topic, desire to participate in science, and personal enjoyment [14,29]. Naturally, the desire to increase their knowledge about beans and recipes using beans was a big pull for this project. Moreover, a major theme under ‘Values’ was volunteer spirit. Several citizen scientists were already active volunteers and/or had experience participating in other citizen science projects, suggesting the importance of positive prior experiences to encourage continued participation in public science. The Bean Cuisine was also uniquely positioned to contribute to participant health and cooking skills, and many participants expressed a desire to learn about how to incorporate more beans in their diets as a way to directly improve their health or that of their loved ones. Clearly, people recognize the importance of pulses in a healthy diet, but they do not always appear to feel confident about how to prepare them in versatile, delicious ways that foster regular inclusion in the diet. In the ‘Interests’ theme, many stated a direct interest in beans and/or cooking, which would be expected due to the tendency of citizen scientist participants to already have an established interest in the project topic [14].

As demonstrated in Table 4, citizen scientists reported various knowledge gains due to participation, which matches the significant increases in knowledge of pulse health benefits, versatility, and cooking of dry pulses shown in Table 2. Participants also frequently commented on the surprising versatility of pulses, demonstrating an increased awareness of ways to use them in the kitchen. Importantly, not only did they find new favorite recipes, but they recognized ways to easily incorporate pulses into their current diets. For example, after realizing that pulses work well in smoothies and with roasted vegetables, participants can easily include pulses without having to learn completely new base recipes. Instead, they can make a quick modification to their dietary patterns with a simple addition of pulses to currently preferred foods (e.g., smoothies, roasted vegetables, oatmeal). Even those who reported loving beans before beginning explained how the recipes encouraged them to think outside of the box and “got us out of our ruts”. Liking for Mayocoba beans, one of the beans sent to citizen scientists in their toolkit, was also commonly expressed. Many individuals had not previously tried this bean but stated an intention to now often include it in their diets, showing the potential of the introduction of new pulses to generate interest and excitement.

Citizen scientists also reported an overall positive experience and expressed a desire to continue participating in citizen science efforts. This is likely due to the fun they had, the knowledge and skills they gained that they deemed useful, and the sense of fulfillment they felt due to their meaningful contribution, e.g., “This was a fun way to participate in an interesting project that I think could really help others.”

A highly encouraging finding was the role citizen scientists adopted in their communities of sharing project knowledge and being pulse advocates. Other citizen science projects have also found that citizen scientists exhibit behaviors suggesting they act as program advocates [39,40]. Citizen science is suggested to provide deeper meaning to participants’ interests and hobbies [41]. In this sense, participation in the Bean Cuisine project could equip participants with more topic area knowledge, inspire them to explore pulse versatility, and further their interest, which naturally leads to them sharing with others. In this study, participants shared with family, neighbors, and friends, including everyone in recipe feedback. This provided greater depth of feedback, as well as creating an opportunity for shared learning about the benefits and versatility of pulses.

In this analysis of the outcomes of participation, it is evident that the benefits for citizen scientists went beyond mere knowledge gain, and cooking and consumption behavior was also impacted. This is critical because although knowledge is important, knowledge in and of itself does not successfully drive behavior change. To achieve the true adoption of healthful behaviors, it is essential to increase knowledge, motivate individuals (e.g., through participation in the project and engagement with scientists and like-minded individuals), and encourage behavioral skills (e.g., tips to cook dry pulses, preparing pulses in versatile ways) [11].

### 4.6. Sensory Panel

Overall, sensory panelists reported being satisfied with all the dishes (see Table 5). The recipe that ranked most highly was the White Bean Waldorf Salad, demonstrating the potential to increase bean consumption by adding beans to dishes people already eat, but may not think to include beans. Indeed, one sensory panelist stated, “I didn’t think beans belonged in Waldorf, but they DO.” Promoting creative ways to eat more pulses, especially when it only requires the simple addition of pulses to a dish people already include in their daily lives rather than learning a whole new recipe, is one approach to quickly increase pulse intake.

### 4.7. The Bean Cuisine as a Model for Stealth Health Approaches

The Bean Cuisine serves as a pilot study for future citizen science projects with broader public health goals. This work demonstrated the feasibility of actively engaging the public in similar projects that can directly improve dietary habits. Participating as citizen scientists provided an extra incentive to try new recipes and eating habits and to begin routinizing healthier dietary habits, in this case through higher pulse inclusion in the diet. Thus, citizen science efforts represent a powerful opportunity to improve public well-being—and even influence positive environmental outcomes when the food is associated with planetary benefits, as is the case with pulses [3].

Adoption of dietary patterns similar to the Bean Cuisine would reverse the current fiber gap (i.e., the dramatic difference between recommended and actual fiber intake) faced in the United States and many countries around the world [42]. Also, it would help promote adequate consumption of other dietary components of public health concern, like potassium [8]. As we increasingly face public and environmental health challenges, it is important to recognize the underutilized power in citizen science approaches and take better advantage of engagement research to shift dietary patterns for the public good.

### 4.8. Limitations and Future Directions

There are several key limitations to this study. As previously explained, there was a relative lack of sociodemographic diversity among participants, which may limit generalizability to a wider audience in aspects such as taste preferences. Also, those who are already interested in the topic—and therefore likely have greater baseline knowledge and potentially greater consumption levels of pulses—were more likely to participate. Again, this poses a challenge when considering the feasibility of this type of citizen science and stealth health approach on a broader audience. Due to the nature of the study and data collection tools, it was not possible to examine in-depth changes to pulse preparation and consumption habits, nor longer-term behavior changes or overall health impacts. Future studies could engage a more diverse group of citizen scientists to actually test the whole Bean Cuisine rather than providing feedback on one day of recipes. Ideally, this type of study would also collect data on health markers (e.g., stool samples to assess changes to the gut microbiome [43]) and longer-term impacts on behavior change, for instance, checking if pulse consumption remains high at several time points after completion of the project. Another measurement of interest would be any effects on intestinal discomfort [44], as concerns over flatulence pose a barrier to higher pulse consumption [11] and addressing these could be helpful in promoting increased intake. Research suggests that many individuals do not experience increased intestinal discomfort with beans and that most of those who do have discomfort see symptoms dissipate within one to three weeks [44]. Testing this finding, especially considering higher pulse intake levels than in previous studies, would be informative about the likely willingness of people to adopt such a dietary pattern. Lastly, the Bean Cuisine could be expanded, incorporating a wider variety of recipes from countries around the globe—including India, Japan, Ethiopia, Mexico, and others—to better represent and showcase the diverse culinary uses of pulses. Drawing inspiration from countries with higher pulse intake, such as Rwanda and Burundi [10], could also help inspire ways to better routinize regular pulse consumption.

## 5. Conclusions

Increasing the production and consumption of beans and other pulses can provide a wide array of human and environmental health benefits, all at a relatively economical price. Despite the impressive list of benefits that could be reaped through the higher incorporation of pulses into daily diets, consumption is low in many countries around the world, with one of the main barriers being a lack of familiarity with how to prepare pulses and take advantage of their versatile uses. Citizen science provides a unique approach to advancing consumption because it actively engages the public and addresses this barrier to consumption, empowering participants to be advocates for the benefits of pulse consumption within their own communities. Citizen scientists reported increases in pulse intake, usage of dry pulses, and knowledge about pulse benefits and versatility, and they explicitly stated their intentions to regularly eat pulses. This highlights how this type of outreach research can increase public knowledge and move the needle for the adoption of healthy eating behaviors, for instance, by helping routinize higher pulse consumption through a stealth health approach. Moreover, citizen scientists engaged in spreading awareness about the project and pulses, becoming stronger pulse advocates in their communities. Thus, the presented results suggest that involving the public in the research process is mutually beneficial to scientists and the public alike, broadening reach and impact beyond the doors of academic institutions to engage and benefit a wider audience. Increases in knowledge and engagement with the scientific community can be perceived as benefits for citizen scientists. However, citizen science projects are also positioned to move beyond mere increases in knowledge level to help attain participant adoption of healthy dietary patterns. To strengthen the ability of citizen science research to draw conclusions about the influence of participation upon behavior changes—such as significant impacts on pulse consumption—it is critical to design sensitive measures. Doing so will be essential to support increased recognition of the potential of citizen science to move the needle on behavior change that advances public and environmental health.

## Figures and Tables

**Table 1:** Average scores of the original Bean Cuisine recipes.

	Recipe Easy to Understand	Ingredients Easy to Find	I Would Make This Recipe Again	Taste	Texture	Appearance	Overall Acceptability
Average score ^1^	4.8	4.6	3.8	4.0	4.1	4.2	4.0
Minimum score ^2^	4.0	3.0	1.5	2.25	1.5	2.0	2.0
Maximum score	5.0	5.0	5.0	5.0	5.0	5.0	5.0
Range	1.0	2.0	3.5	2.75	3.5	3.0	3.0

^1^ A 1 represents a low (poorly rated) score, and 5 a high score. ^2^ Considering the average scores for these 7 components (i.e., the total score for a recipe when averaged for the 4 individuals providing feedback), the minimum and maximum scores reflect the lowest and highest scores for the 56 recipes.

**Table 2:** Pulse knowledge before and after being a Bean Cuisine citizen scientist.

Knowledge of Pulse Nutrition and Health Benefits	Pre: *n* (%)	Post: *n* (%)
1 (low)	2 (3.6)	0
2	4 (7.1)	0
3	10 (17.9)	5 (8.9)
4	24 (42.9)	30 (53.6)
5 (high)	16 (28.6)	21 (37.5)
Average score	3.86	4.29
Difference (*p*-value)		0.43 (0.002)
**Knowledge of Pulse Versatility**	**Pre: *n* (%)**	**Post: *n* (%)**
1 (low)	2 (3.6)	0
2	7 (12.5)	1 (1.8)
3	24 (42.9)	4 (7.1)
4	13 (23.2)	24 (42.9)
5 (high)	10 (17.9)	27 (48.2)
Average score	3.39	4.38
Difference (*p*-value)		0.99 (<0.001)
**Knowledge of How to Prepare Dry Pulses**	**Pre: *n* (%)**	**Post: *n* (%)**
1 (low)	2 (3.6)	1 (1.8)
2	8 (14.3)	1 (1.8)
3	15 (26.8)	4 (7.1)
4	19 (33.9)	21 (37.5)
5 (high)	12 (21.4)	29 (51.8)
Average score	3.55	4.36
Difference (*p*-value)		0.81 (<0.001)

**Table 3:** Motivation to participate in the Bean Cuisine citizen science project.

Overarching Theme	Sub-Theme	Citizen Scientist Quotes
**Learning**	Want to learn about beans Want to learn new, tasty recipes (for themselves and others) Want to learn more ways to include beans in their diet	“I’m interested in trying new recipes and adding legumes to my diet on a daily basis.” “I have two main interests in participating: to help with the research and to find more creative ways to use beans for preparing meals.” “I am really interested in increasing my beans/legumes knowledge through this CSU research opportunity and hope to share it with family and friends.” “I eat a lot of beans and would like to find some more recipes and some other beans or pulses I have never tried.”
**Health Concerns**	Personal or family health concerns Want to adopt a more plant-based diet Desire for affordable healthy food	“I am interested in expanding my diet, more so towards local foods, vegetarian or low meat options, and plant-forward recipes. This seemed like a great opportunity to do a lot of those things!” “Improve fiber in my diet.” “I’m determined to get myself into remission or better.” “My [family member] has high blood pressure, so any opportunity to learn more about nutrition and cooking is one I welcome.” “I am always looking for ways to make our diet healthier.” “I watch my grocery budget. Beans/pulses are affordable, nutritious and healthy, and also provide protein in my largely vegetarian diet.”
**Values**	Volunteer spirit Support local food systems Like encouraging others to eat healthfully	“I love participating as a volunteer.” “I have participated as a citizen scientist… for the last three years. It has been fun and enlightening.” “I am a huge supporter of Colorado Proud and love using my own organic ingredients and other locally-grown products!” “I am passionate about encouraging others to cook healthy, nourishing food at home.”
**Interests**	Beans Cooking Gardening Science	“I love beans! I am a registered dietitian and former recipe developer, so this project is of interest to me.” “I love to garden and eat! I especially love to cook beans and am always excited about trying new recipes.” “I’m a bean lover who wants to get others as excited as I am about this wonderful food source. Plus, science is super fun.” “I enjoy cooking and like to try different recipes.”
**Enjoyment**	Fun Like being a part of a project Enjoy research and science	“This sounds fun; I’d love to be a part of it!” “We like research projects.” “I enjoy participating in research projects, especially food safety and nutrition-related research.”

**Table 4:** Themes of citizen scientist overall experience in the Bean Cuisine.

Theme	Sub-Theme	Example Quote
**Knowledge of Pulses**	Learn about beans Health benefits Ways to eat more beans Bean/pulse cooking	“It was fun to try new recipes and learn about an ingredient I don’t use all that much.” “I also learned more about the health benefits of beans, as well as the classifications of beans and legumes and pulses.” “I really enjoyed doing this! It was great fun to try new recipes and learn more about how to incorporate beans into our daily meals.” “I really enjoyed this project, and am happy that I participated as it expanded my knowledge of how to make and use pulses in a variety of ways.” “I really was surprised about the versatility of the pulses. I like to experience different recipes but would never have thought about making smoothies with them or pairing lentils with shrimp. Making the beans and rice salad was also a pleasant surprise.” “Also, that guide to how to cook dry pulses is amazing and so useful. I’ve cooked with dry beans many times before but less thoughtfully, and the guide did make them taste better.” “Probably the best takeaway for me has been learning how easy it is to cook dry beans in a slow cooker! Thank you very much!”
**Awareness of Pulse Variety**	Experimentation with new types of beans	“I really like the Mayocoba beans! I had not cooked with them before, and for other recipes that need a bean that keeps its shape… this is the perfect bean!” “Thank you for introducing me to Mayocoba beans. I really like them now!” “The recipe was delicious! My first time having Mayocoba beans; wow, love them!”
**Awareness of Pulse Versatility**	Incorporation of beans into their current routine Increased awareness of recipes and ways to use pulses	“This was so simple and straightforward to make. I roast veggies regularly and, from here on, will add some beans to match whatever flavor profile I’m cooking (white beans with roasted Brussels sprouts or cabbage, etc.). Simply genius way to get more beans into a meal!” “I make smoothies every morning. Now that I have made this recipe with beans, I use any bean I have in the fridge as leftovers and use them in my fruit/veggie smoothies.” “The Mayocoba beans were a very nice flavor in the smoothie, preferable to the organic, no-sugar protein powder I have been using. Thank you for the introduction to this change!” “I think it was fun, and I definitely have a couple of new recipes to play around with. My favorite new idea is mixing the beans into baked oats.” “It was really fun trying new recipes! We are vegans who love beans, but the recipes got us out of our ruts.”
**Pulse Consumption Behavior**	Eating more pulses Adding to recipe rotation	“I’m more aware of ways to incorporate beans into every meal, and I’m paying more attention and trying to eat them more.” “I have been eating about one cup of beans every day! And I feel like my intestines are liking this new addition!” “I’m going to start eating more pulses:-)” “Definitely will integrate more pulses into my daily cooking.” “This was really good. My kids, 11 and 13, loved it. It will go into our usual recipe rotation.” “This one was really, really nice. I’ve enjoyed all the recipes so far, and I will probably use them again, but this one is for sure going into the rotation. It hits the golden ratio of ease in preparation, high veggie content and tastiness—while also making enough for two meals for my household!” “The recipe was delicious. Since we eat oats every morning, we will be enjoying this recipe often.” “I loved this recipe! It is great for my gluten-free friends and is really tasty and moist. I will definitely make it again soon!”
**Pulse Cooking Skills and Habits**	How to cook dry beans Experimentation with pulse ingredients Purchasing intent Repurposing of leftovers	“I usually use canned pulses, but I am now more experienced with dried, and they are much tastier, so I look forward to using dried pulses more in the future.” “It was great to learn of different ways to cook pulses, and I’ll likely try using more dried ones than canned ones in the future (even though it’s so easy to open a can!).” “It was fun to try a new recipe that encouraged me to stretch my skills and use dry pinto beans in a recipe.” “I am now experimenting with chickpea flour and including pasta made with lentils in my recipes.” “My first time cooking with Mayocoba beans, and I really like them! I will seek them out in the future.” “I now love Mayocoba beans and will be looking for them.” “We warmed up the leftovers on a cast iron skillet with butter and topped with a drizzle of maple syrup. It was good.”
**Other Personal Outcomes**	Enjoyment Fulfillment	“It’s been fun to try out recipes. The beans sent are fresh and tasty.” “It has been very fun to participate in this research AND try new bean recipes!” “This was a fun way to participate in an interesting project that I think could really help others. I learned some new recipes, and we look forward to seeing the finished project.”
**Perceptions of Citizen Science**	Fun Would participate in citizen science again Meaningful	“Citizen science is fun!” “This was a great experience, and I will be looking to do more as a citizen scientist.” “Being a ‘citizen scientist’ is a wonderful way for a broad group of people from different backgrounds to provide their input, experience and suggestions and collect a large dataset which otherwise may be difficult to obtain.” “I really enjoyed participating in this project. I think it is a super-creative way to raise awareness about food and nutrition, and it’s very meaningful to the participants.”
**Engagement and Advocacy**	Engaging others in feedback Sharing knowledge and recipes with others Pulse advocacy	“I made the snickerdoodle hummus and the chicken and bean cassoulet for friends. We all enjoyed tasting them and giving feedback. I wish I had made the chickpea Dutch baby for guests. It looked like something out of a gourmet magazine.” “This recipe additionally got the approval of two football fans watching Monday night football, and they now know what Mayocoba beans are!” “My mother-in-law was visiting us, and she really enjoyed this salad, saying it tasted really good and healthy! “I shared the finished product with my neighbor, and she liked the different recipes also.” “All positive comments from the taste testers here.” “I don’t eat a lot of sweets, so I brought it to a… happy hour. It was a hit. The beans are a secret ingredient. No one knew beans were included until I told them. Also, no one was familiar with Mayocoba beans, and these were a bunch of gardeners!” “I served a salad at a family get-together. Everyone loved it, even my 4 and 5 year-old grandchildren.” “I served this recipe to a friend who is vegan and also limits her intake of oils. I served it with fresh veggies (red peppers and cucumbers), and it was a big hit with my friend as well as non-vegan guests.” “These were delicious, and my kids happily ate them!” “This was so much fun. I was glad to share them with my husband, and he liked all of them. He will be making the oatmeal and Waldorf salad in the future for sure!” “The instructions should start with soaking and cooking instructions so it’s easily shared!” “This also encouraged me to add more legume talk to my nutrition education at work.”

**Table 5:** Sensory panel feedback.

Dish Name	Meal	Logic for Including in the Sensory Panel	Quotes About the Dish	Average Overall Acceptability Score ^1^	Minimum—Maximum (Range)
Strawberry Banana Bean Smoothie	Breakfast	A smoothie gets people thinking outside of the box and in the realm of beans for breakfast. It is also fast and easy to make, improves the healthiness of smoothies, and is versatile because a wide variety of beans can be used in this recipe.	“Interesting combination. A fascinating way to increase protein and fiber in this product.” “Great dish, tastes amazing! Unbelievable that it contains beans!”	4.5	4–5 (1)
White Bean Waldorf Salad	Lunch	Bean salads are quick recipes and make good leftovers to keep on hand. This recipe also showcases adding beans to a dish people may not originally think to add them to, encouraging them to do this with other dishes as well.	“I very much appreciate adding the nutrition benefits of beans to a classic; it enhances flavor and nutrition.” “This is so good. I would eat this for three meals a day. I didn’t think beans belonged in a Waldorf, but they DO.”	4.75	4–5 (1)
Olive Bean Dip	Snack	This is a very simple, quick dip that can be made with pantry staples and fits into most schedules. It is flexible and can be prepared with almost any bean.	“I think this is one of the most easily acceptable dishes based on how easily it can be introduced into the regular diet.” “Would love to see some fresh greens or chunks of olives.”	4.18	3–5 (2)
Turkey and Bean Skillet	Dinner	A recipe that demonstrates adding beans to a meat dish to boost nutrition and fiber and can also help cut food costs.	“Very good as is—I would add tomatoes for acid, liquid and color.” “Small preference things here—more color, texture, and spice.”	4.17	1–5 (4)

^1^ A score of 5 indicates extremely satisfied and 4 somewhat satisfied. Overall acceptability scores for all the dishes fell within this range and were ranked as satisfying by panelists.

## Data Availability

Data are contained within the article or Supplementary Material.

## Supplementary Materials

The following supporting information can be downloaded at: https://www.mdpi.com/article/10.3390/foods12142667/s1, Supplementary Materials S1: Nutritionist Pro nutrient analysis output of the original 14 day Bean Cuisine; Supplementary Materials S2: Bean Cuisine meals report; Supplementary Materials S3: citizen science intake form; Supplementary Materials S4: recipe feedback form; Supplementary Materials S5: citizen science demographics; Supplementary Materials S6: Nutritionist Pro nutrient analysis output of the updated Bean Cuisine.
