# Supplementary material for: Bean Cuisine: The Potential of Citizen Science to Help Motivate Changes in Pulse Knowledge and Consumption
Source: Foods. 2023 Jul 11;12(14):2667. doi: 10.3390/foods12142667 (PMC10379374; doi:10.3390/foods12142667)
Supplement: Supplementary file 1 [file foods-12-02667-s001.zip › Supplementary Materials S1 - Original Bean Cuisine - Nutrient Analysis.pdf]

Menu Template Nutrient Analysis

Total Days: 14
Total Foods: 151

Avg. Daily Kcals: 1999.343
Name: Original Bean Cuisine

Nutrient	Value	Unit	Goal	%
Weight	2143.808	g		
Kilocalories	1999.343	kcal	2000.000	100 %
Kilojoules	8262.630	kj		
Protein	95.639	g	50.000	191 %
Carbohydrate	291.866	g	275.000	106 %
Available Carbohydrate	0.017	g		
Fat, Total	58.682	g	78.000	75 %
Alcohol	0.249	g		
Cholesterol	209.187	mg	300.000	70 %
Saturated Fat	14.324	g	20.000	72 %
Monounsaturated Fat	17.924	g		
Polyunsaturated Fat	13.644	g		
SFA 4:0	0.225	g		
SFA 6:0	0.151	g		
SFA 8:0	0.094	g		
SFA 10:0	0.219	g		
SFA 12:0, Lauric	0.238	g		
SFA 14:0	0.767	g		
SFA 15:0 Pentadecanoic acid	0.039	g		
SFA 24:0 Lignoceric Acid	0.003	g		
SFA 16:0, Palmitic	6.742	g		
SFA 17:0	0.052	g		
SFA 18:0, Stearic	2.286	g		
SFA 20:0	0.041	g		
SFA 22:0	0.014	g		
MFA 14:1	0.032	g		
MFA 16:1	0.481	g		
MFA 18:1, Oleic	16.754	g		
MFA 20:1	0.160	g		
MFA 22:1	0.010	g		
PFA 18:2, Linoleic	11.382	g		
PFA 18:3, Linolenic	1.799	g		
PFA 18:4	0.004	g		
PFA 20:4	0.104	g		
PFA 20:5, EPA	0.010	g		
PFA 22:5	0.008	g		
PFA 22:6, DHA	0.041	g		
Trans Fatty Acid	0.328	g		
Sum of Trans Fat and Sat Fat	9.876	g		
Omega 3 Polyunsat Fat, Tot	0.000	g		
Omega 6 Polyunsat Fat, Tot	0.000	g		
Sodium	1933.959	mg	2300.000	84 %
Potassium	5142.688	mg	4700.000	109 %
Salt	4.838	g		
Chloride			2300.000	
Vitamin A (RE)	1658.216	RE		

Nutrient	Value	Unit	Goal	%
Cobalamin (Vitamin B12)	6.261	mcg	2.400	261 %
Biotin	21.298	mcg	30.000	71 %
Pantothenic Acid	5.505	mg	5.000	110 %
Vitamin K	267.241	mcg	120.000	223 %
Phosphorus	1524.381	mg	1250.000	122 %
Iodine		mcg	150.000	
Magnesium	498.766	mg	420.000	119 %
Zinc	10.911	mg	11.000	99 %
Copper	1.978	mg	0.900	220 %
Manganese	4.759	mg	2.300	207 %
Selenium	81.934	mcg	55.000	149 %
Fluoride	75.690	mcg		
Chromium	0.048	mg	0.035	138 %
Molybdenum	40.987	mcg	45.000	91 %
Choline	372.470	mg	550.000	68 %
Chlorine		mg		
Sulfur		mg		
Cobalt		mcg		
Boron		mcg		
Nickel		mcg		
Dietary Fiber, Total	61.469	g	28.000	220 %
Soluble Fiber	2.762	g		
Insoluble Fiber	4.571	g		
Crude Fiber	19.461	g		
Sugar, Total	88.943	g		
Added Sugars	11.893	g	50.000	24 %
Glucose	19.790	g		
Galactose	1.262	g		
Fructose	21.934	g		
Sucrose	20.247	g		
Lactose	7.737	g		
Maltose	0.863	g		
Sugar Alcohol	0.000	g		
Other Carbohydrate	0.000	g		
Sorbitol	0.000	g		
Xylitol	0.000	g		
Inositol		mg		
Tryptophan	848.866	mg		
Threonine	2911.142	mg		
Isoleucine	3269.393	mg		
Leucine	5749.628	mg		
Lysine	5077.066	mg		
Methionine	1354.678	mg		
Cystine	869.929	mg		
Phenylalanine	3636.166	mg		
Tyrosine	2320.688	mg		
Valine	3931.118	mg		
Arginine	4561.384	mg		

Nutrient Goal Template
DAILY VALUES/RDI - ADULT/CHILD

Analyzed by
Original Bean Cuisine

Exchanges

Bread/Starch	2.00
Fat	0.50
Fruit	3.00
Meat-High Fat	0.00
Meat-Medium Fat	0.00
Meat-Very Lean	0.00
Milk-Skim	0.50
Other Carbohydrate	0.00
Vegetables	1.00

Menu Template Nutrient Analysis

Total Days: 14
Total Foods: 151

Avg. Daily Kcals: 1999.343
Name: Original Bean Cuisine

Nutrient	Value	Unit	Goal	%
Vitamin A (IU)	14800.530	IU	5000.000	296 %
Vitamin A (RAE)	1866.178	mcg	900.000	207 %
Total Carotenoid	0.000	RE		
Beta-Carotene	8930.838	mcg		
Alpha-Carotene	1732.957	mcg		
Lutein (+ Zeaxanthin)	4636.036	mcg		
Beta-Cryptoxanthin	187.945	mcg		
Lycopene	7268.183	mcg		
Vitamin C	193.251	mg	90.000	215 %
Calcium	1450.116	mg	1300.000	112 %
Iron	20.203	mg	18.000	112 %
Vitamin D (ug)	7.556	mcg	20.000	38 %
Vitamin D (IU)	304.746	IU	400.000	76 %
Vitamin E (mg)	0.269	mg	20.000	1 %
Vitamin E (IU)	0.401	IU	30.000	1 %
Vitamin E (Alpha-Tocopherol)	10.357	mg	15.000	69 %
Beta Tocopherol	0.098	mg		
Gamma Tocopherol	2.232	mg		
Delta Tocopherol	0.218	mg		
Alpha Tocotrienol	0.212	mg		
Beta Tocotrienol	0.289	mg		
Gamma Tocotrienol	0.257	mg		
Delta Tocotrienol	0.006	mg		
Thiamin	3.083	mg	1.200	257 %
Riboflavin	3.273	mg	1.300	252 %
Niacin	14.902	mg	20.000	75 %
Niacin Equivalent	32.267	mg	16.000	202 %
Pyridoxine (Vitamin B6)	2.867	mg	1.700	169 %
Folate (Total)	805.451	mcg	400.000	201 %
Folate (DFE)	1010.164	mcg	400.000	253 %
Folic Acid	133.862	mcg		
Food Folate	786.102	mcg		

Nutrient	Value	Unit	Goal	%
Histidine	1994.016	mg		
Alanine	3392.822	mg		
Aspartic Acid	7924.574	mg		
Glutamic Acid	12651.570	mg		
Glycine	2884.937	mg		
Proline	4020.810	mg		
Serine	3746.584	mg		
Glutamine	0.000	mg		
Taurine	0.000	mg		
Hydroxyproline	24.793	mg		
Cysteine	0.000	mg		
Moisture	1427.764	g		
Ash	16.082	g		
Caffeine	3.401	mg		
Osmolality		mo		
Theobromine	30.414	mg		
Betaine	23.225	mg		
Phytosterols	48.141	mg		
Stigmasterol	1.428	mg		
Campesterol	3.595	mg		
Beta-sitosterol	22.840	mg		
Epigallocatechin-3-gallate		mg		
Daidzein	0.015	mg		
Genistein	0.028	mg		
Glycitein	0.000	mg		
Total isoflavones	0.050	mg		
Biochanin A	0.089	mg		
Formononetin	0.014	mg		
Coumestrol	0.001	mg		
