# Supplementary material for: Bean Cuisine: The Potential of Citizen Science to Help Motivate Changes in Pulse Knowledge and Consumption
Source: Foods. 2023 Jul 11;12(14):2667. doi: 10.3390/foods12142667 (PMC10379374; doi:10.3390/foods12142667)
Supplement: Supplementary file 1 [file foods-12-02667-s001.zip › Supplementary Materials S2 - Bean Cuisine - Meals Report.pdf]

Menu Template Meals Report

Total Days: 14
Total Foods: 151

Avg. Daily Kcals: 1999.343
Name: Original Bean Cuisine

	Breakfast	Lunch	Dinner	Morning Snack	Afternoon Snack	Evening Snack
Day 1	Chickpea Flour Scramble	Multi-Bean Salad	Zesty Salsa Chicken Pulse Pasta		Olive Bean Dip	
	Avocado, California	Orange	Garden Salad		WHEAT THINS Crackers, Hint of Salt	
	Yogurt, Plain, Low Fat (12 grams protein per 8 ounces)	PLANET OAT Milk, Oatmilk, Original			SILK Plus Omega-3 DHA, soymilk	
	Banana				DOVE PROMISES Candy Bar, Dark Chocolate, Silky Smooth	
	Milk, Low Fat, 1%					
Day 2	Chocolate Black Bean Smoothie	Bean and Tuna Salad Sandwich	Bean and Quinoa Harvest Salad		Crispy Chickpeas	
	Eggs, Hard Boiled	Carrots, Baby	Blueberries		Almonds, Dry Roasted, without Salt Added	
		SILK Plus Omega-3 DHA, soymilk			PLANET OAT Milk, Oatmilk, Original	
					Dates, Medjool	

Menu Template Meals Report

Total Days: 14
Total Foods: 151

Avg. Daily Kcals: 1999.343
Name: Pulse Cuisine_v1_ORIGINAL

	Breakfast	Lunch	Dinner	Morning Snack	Afternoon Snack	Evening Snack
**Day 3**	Apple Cinnamon Lentil Porridge	Bean and Cheese Burrito	Ginger Chicken and Beans		Black Bean Brownie	
	Yogurt, Plain, Low Fat (12 grams protein per 8 ounces)	Watermelon	Rice, Brown, Medium Grain, Cooked		PLANET OAT Milk, Oatmilk, Original	
	Strawberries (Strawberry)	Juice, Lime	Grapes, Red or Green			
**Day 4**	Carrot Cake White Bean Oatmeal	White Bean Waldorf Salad	Bean and Sweet Potato Bake		Savory Chickpea Bread	
	PLANET OAT Milk, Oatmilk, Original	Garden Salad with Chicken	Eggs, Fried		Yogurt, Plain, Low Fat (12 grams protein per 8 ounces)	
		Orange			Blueberries	
**Day 5**	Easy Baked Beans on Toast	Rocky Mountain "Caviar"	Lentil Curry		Buffalo Chickpea Dip	
	Orange	FOOD SHOULD TASTE GOOD Chips, Tortilla, Multigrain	Broccoli		Celery, Stalk	
	Milk, Low Fat, 1%	Apple, Medium	Strawberries (Strawberry)		Carrots	

Menu Template Meals Report

Total Days: 14
Total Foods: 151

Avg. Daily Kcals: 1999.343
Name: Pulse Cuisine_v1_ORIGINAL

	Breakfast	Lunch	Dinner	Morning Snack	Afternoon Snack	Evening Snack
			SILK Plus Omega-3 DHA, soymilk		Dates, Medjool	
Day 6	Chickpea Flour Everything Bagel Muffins	Black Bean Salad	Bean and Turkey Quesadilla		Peanut Butter White Bean Blondie	
	Grapefruit, Pink or Red	Eggs, Hard Boiled	Salsa		PLANET OAT Milk, Oatmilk, Original	
	SILK Plus Omega-3 DHA, soymilk	Kiwi Fruit, Green or Chinese Gooseberries				
Day 7	Pinto Bean Shakshuka	Herby Bean Toast	White Bean Chicken Chili		Artichoke Bean Dip	
	Bread, Pita, Whole Wheat	Garden Salad	Orange		Pepper, Bell or Sweet, Red	
	Yogurt, Plain, Low Fat (12 grams protein per 8 ounces)	PLANET OAT Milk, Oatmilk, Original			DOVE PROMISES Candy Bar, Dark Chocolate, Silky Smooth	
					Cucumber	
Day 8	Strawberry Banana Bean Smoothie	Bean and Rice Salad	Lentil, Broccoli, and Shrimp Bake		Pinto Pumpkin Dip	

Menu Template Meals Report

Total Days: 14
Total Foods: 151
Avg. Daily Kcals: 1999.343
Name: Pulse Cuisine_v1_ORIGINAL

	Breakfast	Lunch	Dinner	Morning Snack	Afternoon Snack	Evening Snack
	Peanuts, dry roasted, without salt	SILK Plus Omega-3 DHA, soymilk<br>Dates, Medjool	Potatoes, Sweet, Baked in Skin<br>Cottage Cheese, Low Fat 1%<br>Chives, Chopped		Pepper, Bell or Sweet, Green<br>PLANET OAT Milk, Oatmilk, Original	
Day 9	Chickpea Dutch Baby<br>PLANET OAT Milk, Oatmilk, Original	Lentil Tacos<br>Yogurt, Plain, Low Fat (12 grams protein per 8 ounces)<br>Mango	Chicken and Bean Cassoulet<br>Blueberries		Snickerdoodle Hummus<br>Apple, Medium	
Day 10	Black Bean Breakfast Burrito<br>SILK Plus Omega-3 DHA, soymilk	Mediterranean Chickpea Salad<br>Carrots, Baby<br>PLANET OAT Milk, Oatmilk, Original	Pinto and Ham Soup<br>Bread, 100% Whole Wheat		Chocolate Cherry Mayocoba Bean Bars<br>Grapefruit, Pink or Red	

Menu Template Meals Report

Total Days: 14
Total Foods: 151
Avg. Daily Kcals: 1999.343
Name: Pulse Cuisine_v1_ORIGINAL

	Breakfast	Lunch	Dinner	Morning Snack	Afternoon Snack	Evening Snack
Day 11	Savory Chickpea Waffle	Super Simple Pinto Bean Burger	Chicken and Pasta with Beans		Split Pea Hummus	
	Banana	Orange	SILK Plus Omega-3 DHA, soymilk		Cauliflower, Chopped	
	PLANET OAT Milk, Oatmilk, Original				Dates, Medjool	
Day 12	Blueberry Banana White Bean Oats	Beany Salad Wrap	Eggy Lentil Pasta		Mayocoba Cookie Dough Dip	
	PLANET OAT Milk, Oatmilk, Original	Watermelon	Grapes, Red or Green		Crackers, Graham, Plain	
		SILK Plus Omega-3 DHA, soymilk				
Day 13	Chickpea Flour Frittata	Mayocoba Stuffed Sweet Potatoes with Tahini Yogurt Sauce	Turkey and Bean Skillet		Lentil Bruschetta with Tomatoes, Basil, and Feta	
	Grapefruit, Pink or Red	Grapes, Red or Green	Juice, Lime			
	PLANET OAT Milk, Oatmilk, Original	SILK Plus Omega-3 DHA, soymilk				

Menu Template Meals Report

Total Days: 14
Total Foods: 151

Avg. Daily Kcals: 1999.343
Name: Pulse Cuisine_v1_ORIGINAL

	Breakfast	Lunch	Dinner	Morning Snack	Afternoon Snack	Evening Snack
Day 14	Beany Breakfast Hash	Bean and Rice Soup	Summery Split Pea and Tomato Salad		Black Bean Chocolate Mousse	
	Banana	Bread, 100% Whole Wheat	Watermelon		Strawberries (Strawberry)	
	SILK Plus Omega-3 DHA, soymilk				Crackers, Graham, Plain	
					Milk, Low Fat, 1%	
