# Supplementary material for: Bean Cuisine: The Potential of Citizen Science to Help Motivate Changes in Pulse Knowledge and Consumption
Source: Foods. 2023 Jul 11;12(14):2667. doi: 10.3390/foods12142667 (PMC10379374; doi:10.3390/foods12142667)
Supplement: Supplementary file 1 [file foods-12-02667-s001.zip › Supplementary Materials S3 - Bean Cuisine - Citizen Scientist Intake Form.pdf]

Default Question Block

Please complete this citizen science intake form if you would like to participate in our research on developing a Bean Cuisine. Studies from our laboratory suggest that benefits for gut health occur when at least 35% of dietary protein comes from beans and other pulses (e.g., chickpeas, lentils, black-eyed peas, split peas). Thus, we have designed a Bean Cuisine that contains this level of pulses, and we have confirmed in Nutritionist Pro (nutrition software) that it meets dietary guidelines. We would very much value your feedback on recipes and your experience cooking them in your home kitchen. We have approximately 45 spots available and will assign them on a first come, first serve basis, so if this link it still live the spots have not yet been filled. As a citizen scientist, you will be granted access to all the recipes (2 weeks with unique breakfast, lunch, dinner, and snack recipes for each of the 14 days) and asked to provide feedback on 4 recipes by completing a brief feedback form for each one. You will need to purchase the ingredients for the recipes you are assigned, although we do provide Colorado Proud beans and a $20 Amazon gift card to participants as a thank you.

This citizen science project is part of a PhD research project being conducted in the Horticulture and Landscape Architecture and Food Science and Human Nutrition Departments of Colorado State University to conduct outreach efforts that meet needs that consumers express, such as wanting to become more familiar with the diverse ways to prepare beans. It is important to us to engage our communities in research to share what we are learning and also learn from each of you.

Your participation in this project is voluntary, and you may skip any survey question you choose not to answer. You must be 18 or older to participate. Researchers will keep all information confidential. If you have questions, please contact PhD Candidate Chelsea Didinger, at Chelsea.Didinger@colostate.edu, Professor Dr. Henry Thompson, at Henry.Thompson@colostate.edu, or Dr. Marisa Bunning, Extension Specialist and Professor, at Marisa.Bunning@colostate.edu. If you have any questions about your rights as a volunteer in this research, contact the CSU IRB at: RICRO_IRB@mail.colostate.edu; 970-491-1553.

Your input is critical and appreciated. This citizen science project and the associated feedback forms are important to the research project and will allow us to improve upon the Bean Cuisine and learn how to more actively engage our communities. As a thank you for your participation, you:
- Can opt to be mailed Colorado Proud beans
- Will receive a $20 Amazon gift card after you provide feedback on all 4 of the recipes you are assigned

Thank you for your valuable time and input – your contribution makes this research possible!

- ○ YES, I voluntarily agree to participate in this research.
- ○ NO, please exit me from this survey.

Thank you for agreeing to participate. First, we have a few questions about your participation as a citizen scientist.

After completing the brief project training/debrief, do you agree to try to prepare and provide feedback on your 4 recipes within no more than 1 month? Please note that if you do not think this timeline is feasible, we sincerely appreciate your interest but we will have to recruit someone else.

- ○ Yes, I agree
- ○ No, this timeline is not feasible

To make sure everyone understands the project, to provide an opportunity to learn more about our research, and to allow you to ask any questions, we will host 1 live Zoom meeting. We will also provide a link to a recorded debriefing of the project to everyone, but this recording is especially for those who are not available to join the Zoom. Do you agree

that to participate as a citizen scientist, you will either join the Zoom or watch the brief recording?

- ○ Yes
- ○ No

Do you understand and agree that you are responsible for purchasing the ingredients for the recipes? Note that you can request a different recipe if it is not feasible for you to prepare what you were assigned, and that you will receive Colorado Proud beans and a $20 Amazon gift card for participating.

- ○ Yes
- ○ No

If you are willing, please share with us what motivated you to want to participate as a citizen scientist.

On a scale of 1 (low) to 5 (high), how would you rate your current (**before** participating as a citizen scientist) knowledge of the following?

	1 (low)	2	3	4	5 (high)
Knowledge of bean/pulse nutrition and health benefits	○	○	○	○	○
Knowledge of ways to use beans/pulses in various dishes	○	○	○	○	○

	1 (low)	2	3	4	5 (high)
Knowledge of how to prepare dry beans/pulses	○	○	○	○	○

We will assign you 4 recipes to cook. Thus, it is important to understand if you have any food allergies, intolerances, and/or preferences. Of course, if you are somehow assigned a recipe containing ingredients you cannot use - for whatever reason - then you can reach out to us and we will reassign. To lower the possibility of this happening, please provide the following information.

Which of the following **best** describes your dietary pattern?

- ○ Omnivore (you include meat, like chicken, fish, beef, and/or pork)
- ○ Pescatarian (eat eggs, dairy, fish and other seafood, but no chicken, beef, pork, or other animals)
- ○ Vegetarian (no meat, but eat foods such as eggs and dairy)
- ○ Vegan (no animal products, including eggs, dairy, and honey)

Please provide a detailed list of any of your food allergies and/or intolerances. If you have none, please write 'N/A.'

Outside of the dietary pattern and food allergies/intolerances listed above, are there other foods/ingredients we should avoid when assigning you recipes? If yes, please list the foods/ingredients.

- ○ No

- ○ Yes

Now, we would like to ask about your cooking and eating habits. If you participated in our Food Habits Survey, some of these questions may look familiar.

Approximately how often do you eat beans or other pulses? Pulses include chickpeas, lentils, and dry peas like split peas but do NOT include soybeans, peanuts, snap peas, etc. See the graphic below for a more detailed definition of pulses.

- ○ Every day
- ○ 4-6 days per week
- ○ 1-3 days per week
- ○ 1-3 days per month
- ○ Several days per year, but less than 1 day per month
- ○ Never

Pulses are a type of legume that include dry beans like black beans, pinto beans, and kidney beans. Chickpeas, cowpeas (i.e. black-eyed peas), dry peas, and lentils are also pulses. Soybeans and fresh green vegetables such as snap beans and snap peas are NOT considered pulses.

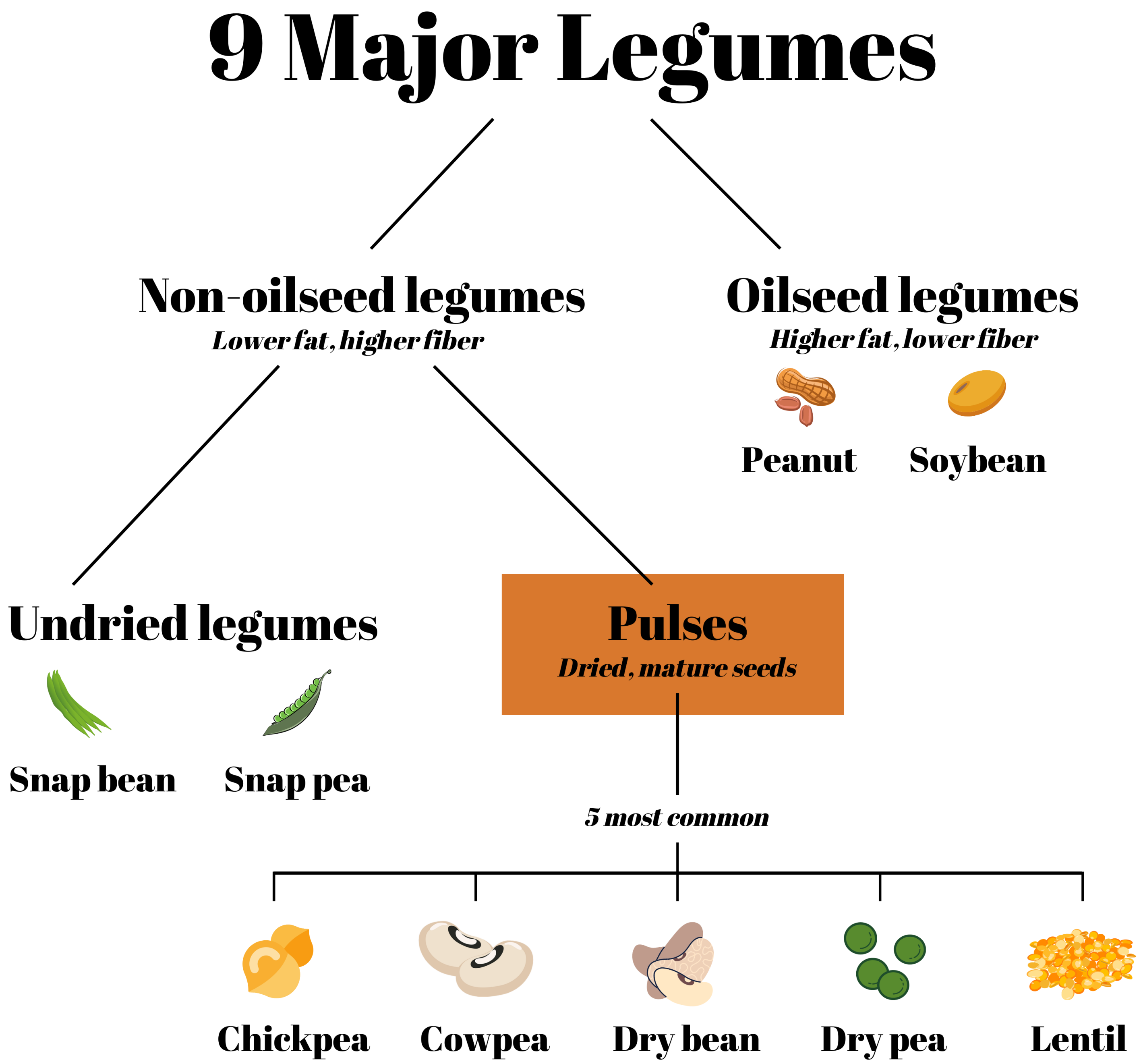

Which do you more frequently cook with, canned pulses or dry pulses you cook yourself?

- ○ Canned
- ○ Dry
- ○ Approximately 50-50

How often do you currently purchase and eat **Colorado-grown** beans?

- ○ Never

- ○ Very rarely (1-20% of the time)
- ○ Somewhat rarely (21-40% of the time)
- ○ Sometimes (41-60% of the time)
- ○ Somewhat often (61-80% of the time)
- ○ Very often (81-100% of the time)
- ○ Unsure if the beans I purchase are from Colorado or not

We will need your contact information to reach out regarding the project and confirm when people complete feedback on all 4 recipes. This information will not be shared outside the research team, and your name will not be associated with any of your responses to other questions.

Full name

First name	
Last name	

Email address - *please double-check that you entered this correctly as correspondence will be conducted via email*

As a thank you for your participation, would you like to be mailed delicious Colorado Proud beans?

- ○ Yes
- ○ No

Please provide your address. We will only use this to mail you Colorado Proud beans and then delete this information.

Street number and name	
City	
State	
Zip code	

Lastly, please answer a few questions about yourself to help us better understand who we are serving.

Gender

- ○ Female
- ○ Male
- ○ Other
- ○ Prefer not to say

In which Colorado county do you live? If you do not currently live in Colorado, please write 'N/A.'

[ ]

Do you identify as Hispanic?

- ○ Hispanic
- ○ Non-Hispanic
- ○ Prefer not to answer

With which ethnicity do you most identify? You can choose multiple options.

- ☐ Asian
- ☐ Black
- ☐ Native American
- ☐ White/Caucasian
- ☐ Other [ ]
- ☐ Prefer not to answer

Age

- ○ 18-20

- ○ 21-29
- ○ 30-39
- ○ 40-49
- ○ 50-59
- ○ 60-69
- ○ 70-79
- ○ 80+
- ○ Prefer not to say

Highest education level

- ○ Some high school, no diploma
- ○ High school graduate or GED
- ○ Some college credit, no degree
- ○ Trade/technical/vocational training
- ○ Associate degree
- ○ Bachelor’s degree, completed or currently enrolled
- ○ Master’s degree, completed or currently enrolled
- ○ Professional degree
- ○ Doctorate degree, completed or currently enrolled
- ○ Other

  ____

- ○ Prefer not to say

Is there anything else you would like to share with us?

Powered by Qualtrics
