# Supplementary material for: Bean Cuisine: The Potential of Citizen Science to Help Motivate Changes in Pulse Knowledge and Consumption
Source: Foods. 2023 Jul 11;12(14):2667. doi: 10.3390/foods12142667 (PMC10379374; doi:10.3390/foods12142667)
Supplement: Supplementary file 1 [file foods-12-02667-s001.zip › Supplementary Materials S4 - Bean Cuisine - Citizen Scientist Feedback Form.pdf]

Default Question Block

Hello, citizen scientist! Thank you for preparing one of the recipes and being ready to provide feedback. Please complete this feedback form once for each of the recipes assigned to you (4 times total, after which you will receive a $20 gift card). Your feedback is critical to the development and improvement of the Bean Cuisine.

As you know, this citizen science project is part of a PhD research project being conducted in the Horticulture and Landscape Architecture and Food Science and Human Nutrition Departments of Colorado State University to conduct outreach efforts that meet needs that consumers express, such as wanting to become more familiar with the diverse ways to prepare beans. It is important to us to engage our communities in research to share what we are learning and also learn from each of you.

Your participation in this project is voluntary, and you may skip any survey question you choose not to answer. You must be 18 or older to participate. Researchers will keep all information confidential. If you have questions, please contact PhD Candidate Chelsea Didinger, at Chelsea.Didinger@colostate.edu, Professor Dr. Henry Thompson, at Henry.Thompson@colostate.edu, or Dr. Marisa Bunning, Extension Specialist and Professor, at Marisa.Bunning@colostate.edu. If you have any questions about your rights as a volunteer in this research, contact the CSU IRB at: RICRO_IRB@mail.colostate.edu; 970-491-1553.

COLORADO STATE UNIVERSITY
EXTENSION

Thank you for your valuable time and input – your contribution makes this research possible!

Are you ready to provide feedback?

- ○ YES, I voluntarily agree to participate in this research.
- ○ NO, please exit me from this survey.

To allow us to track responses submitted, please write your email address.

[ ]

First, we have a few questions about the recipe you prepared.

Of the 4 recipes about which you will provide feedback, what recipe number is this for you?

- ○ First
- ○ Second
- ○ Third
- ○ Fourth

Please confirm that you attended the live Zoom training and/or watched the recorded project debriefing.

- ○ Yes, I attended the live Zoom
- ○ Yes, I watched the recorded project debriefing
- ○ Yes, I attended the live Zoom and watched the recorded project debriefing
- ○ No, I have not yet watched either

Please watch the training before proceeding. After watching, then return and complete this feedback form. Thank you!

Please provide any comments, suggestions, or other feedback about the live Zoom and/or recorded project debriefing. We will only ask this question the first time you complete this form.

Please write the full name of the recipe for which you are providing feedback

To prepare the recipe, did you use canned or dry pulses cooked in the home?
Note: Pulses include dry beans, chickpeas, lentils, and dry peas like split peas but do NOT include soybeans, peanuts, snap peas, etc. See the graphic below for a more detailed definition of pulses.

- Canned
- Dry
- Not applicable - this recipe called for chickpea flour

Pulses are a type of legume that include dry beans like black beans, pinto beans, and kidney beans. Chickpeas, cowpeas (i.e. black-eyed peas), dry peas, and lentils are also pulses. Soybeans and fresh green vegetables such as snap beans and snap peas are NOT considered pulses.

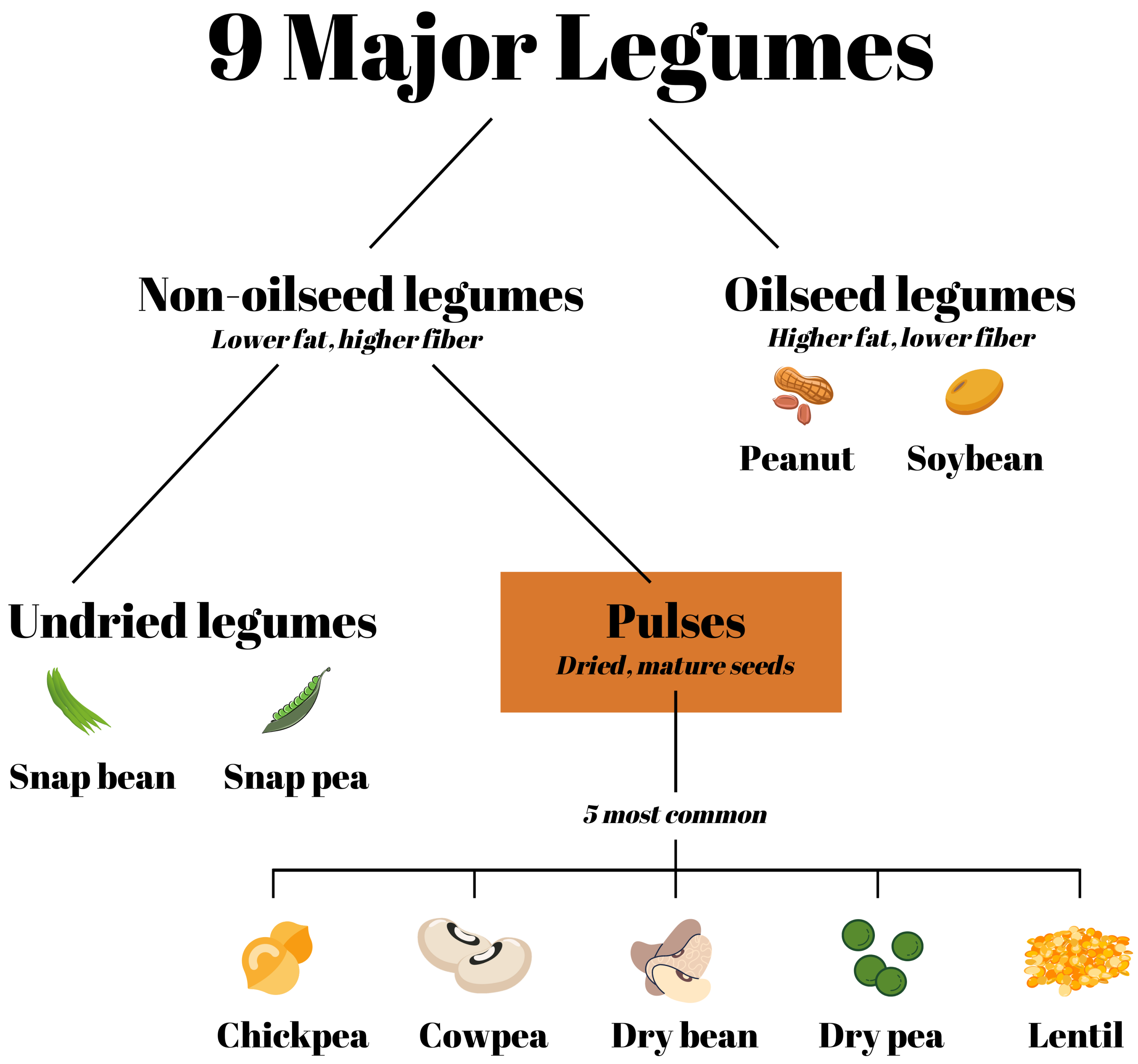

What was the sodium level of the canned pulses?

- ○ No salt added
- ○ Reduced sodium
- ○ Regular

Please share how you cooked the dry pulses.
Did you soak them?

- ○ Yes
- ○ No

Did you add anything to the soaking water? If so, what did you add?

- ○ [ ] Yes
- ○ No

Did you drain the soaking water before cooking?

- ○ Yes
- ○ No

Which of the following did you use to cook the pulses?

- ○ Stovetop
- ○ Electric pressure cooker (like an Instant Pot)
- ○ Slow cooker (like a Crockpot)
- ○ [ ] Other

Approximately how long did it take you **in minutes** to prepare the recipe? (Please do not count the time it took to cook dry pulses, unless that was part of the recipe instructions, like with lentil curry.)

[ ]

Please indicate how much you agree with the following.

	Strongly **agree**	Somewhat **agree**	Neither agree nor disagree	Somewhat **disagree**	Strongly **disagree**
The recipe was easy to understand/follow	○	○	○	○	○
I would make this recipe again	○	○	○	○	○
The ingredients were easy to find	○	○	○	○	○

If you have any recommendations to make the recipe easier to understand/follow, please share them below.

Which ingredient(s) was hard to find?

Now, we would like to ask about your sensory experience.

Please indicate your satisfaction with the overall taste, texture, appearance, and overall acceptability of the finished recipe.

	Extremely **satisfied**	Somewhat **satisfied**	Neither satisfied nor dissatisfied	Somewhat **dissatisfied**	Extremely **dissatisfied**
Taste	○	○	○	○	○
Texture	○	○	○	○	○
Appearance	○	○	○	○	○
Overall acceptability	○	○	○	○	○

Did you prepare the recipe according to the listed ingredients and instructions? If no, please indicate any changes that you made. You can select more than one option.

☐ Yes, I followed the recipe

☐ No, I made substitutions and/or changes to the recipe due to dietary reasons and/or preferences

☐ No, I made substitutions and/or changes to the recipe due to other reasons (could not find ingredients, etc.)

If we were to recommend other pulses people could use in place of the current one(s) listed, what other pulse(s) do you think may work well in this recipe? Please feel free to explain why.

Would you like to recommend any changes to the recipe?

○ Yes

○ No, I recommend leaving it as is

Please explain any changes to the recipe that you would recommend, indicate if you tried these changes, and the tell about the impact on the flavor. (For example: I think the recipe would taste better with fresh lime added at the end. After trying the recipe as is, I squeezed fresh lime over it, and it added a nice zest.)

What changes would you recommend?

Did you try these changes? (yes/no)

If you tried the change(s), how did it impact the flavor?

If you would like to submit a photo of the finished dish, please do so here.

May we use this photo in presentations, papers, and other materials that result from this project? To protect your anonymity, we will only be able to attribute the photo to "a citizen scientist" and will not use your name.

- ○ Yes, you may use it

No, please do not share this outside the research team

Would you like to share anything else about your experience preparing this recipe?

Block 1

Because this is your fourth and final recipe review, we would like to ask you a few questions about your overall experience as a citizen scientist.

As a result of participating in this citizen science project, check the statements with which you agree.

- I am more likely to purchase Colorado-grown pulses
- I now try to eat more pulses
- I learned about the versatility of pulses

On a scale of 1 (low) to 5 (high), how would you rate your current (**after** participating as a citizen scientist) knowledge of the following?

	1 (low)	2	3	4	5 (high)
Knowledge of bean/pulse nutrition and health benefits					
Knowledge of ways to use beans/pulses in various dishes					
Knowledge of how to prepare dry beans/pulses					

Which do you more frequently cook with, canned pulses or dry pulses you cook yourself?

- Canned
- Dry
- Approximately 50-50

Approximately how often do you eat beans or other pulses? Pulses include chickpeas, lentils, and dry peas like split peas but do NOT include soybeans, peanuts, snap peas, etc. See the graphic below for a more detailed definition of pulses.

- Every day
- 4-6 days per week
- 1-3 days per week
- 1-3 days per month
- Several days per year, but less than 1 day per month
- Never

Pulses are a type of legume that include dry beans like black beans, pinto beans, and kidney beans. Chickpeas, cowpeas (i.e. black-eyed peas), dry peas, and lentils are also pulses. Soybeans and fresh green vegetables such as snap beans and snap peas are NOT considered pulses.

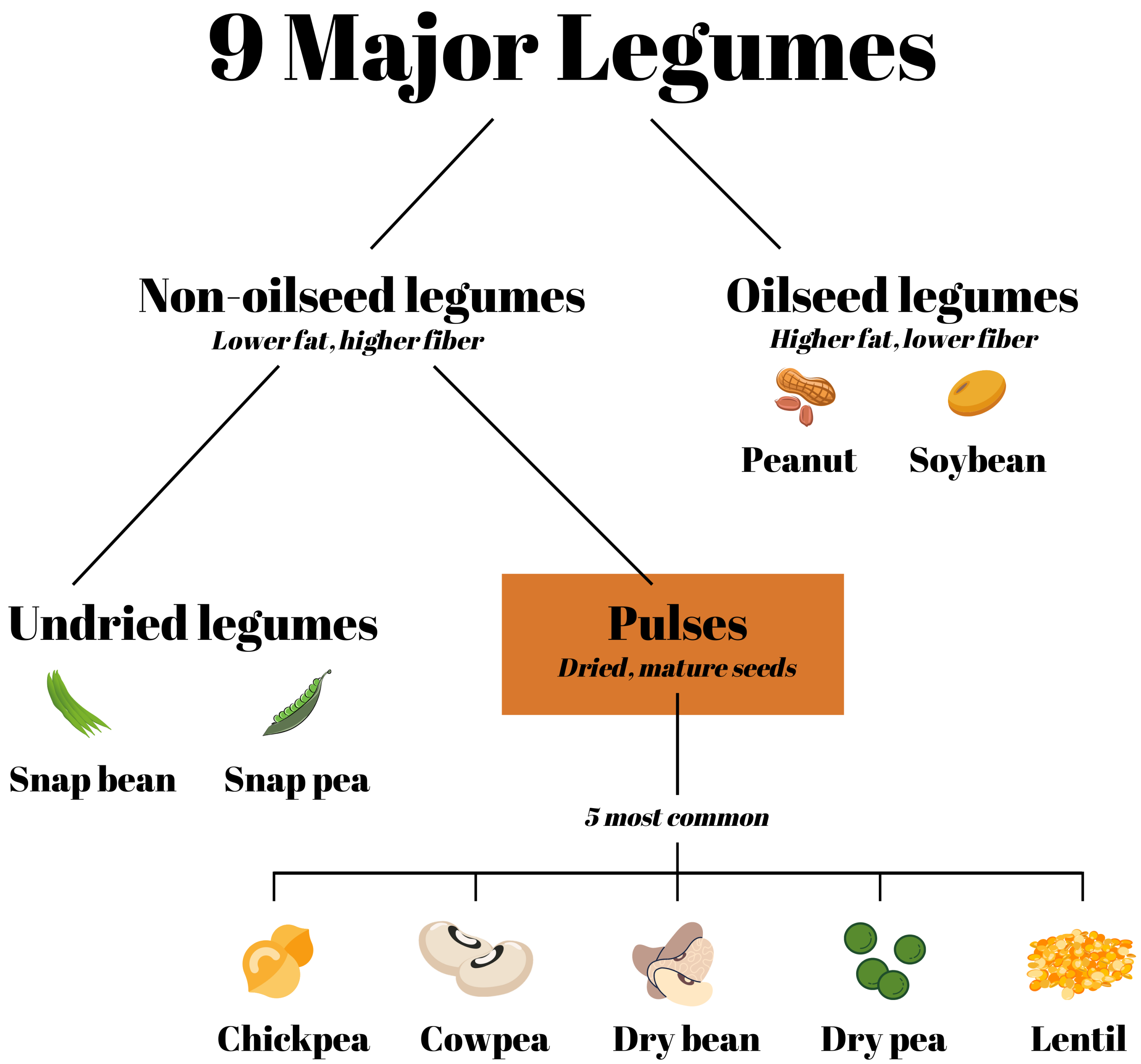

If you are willing, please share with us what about your overall experience as a citizen scientist.

Would you like us to email you the final version of the Bean Cuisine (after we have incorporated changes and finalized formatting)?

- Yes
- No

Powered by Qualtrics
