# Supplementary material for: Bean Cuisine: The Potential of Citizen Science to Help Motivate Changes in Pulse Knowledge and Consumption
Source: Foods. 2023 Jul 11;12(14):2667. doi: 10.3390/foods12142667 (PMC10379374; doi:10.3390/foods12142667)
Supplement: Supplementary file 1 [file foods-12-02667-s001.zip › Supplementary Materials S5 - Citizen Scientist Demographics.pdf]

Dietary Pattern	N (out of 56)	Percent
Omnivore	39	69.6
Pescatarian	5	8.9
Vegetarian	8	14.3
Vegan	4	7.1
**Gender**		
Female	54	96.4
Male	2	3.6
**Age**		
21-29	3	5.4
30-39	4	7.1
40-49	7	12.5
50-59	15	26.8
60-69	19	33.9
70-79	7	12.5
Prefer not to say	1	1.8
**Hispanic**		
Hispanic	1	1.8
Non-Hispanic	55	98.2
**Ethnicity**		
Asian	1	1.8
Native American	1	1.8
Caucasian	53	94.6
Asian-Caucasian	1	1.8
**Highest education level**		
Some college degree, no credit	2	3.6
Trade/technical/vocational training	1	1.8
Associate degree	1	1.8
Bachelor's degree, completed or currently enrolled	24	42.9
Master's degree, completed or currently enrolled	22	39.3
Professional degree	3	5.4
Doctoral degree, completed or currently enrolled	3	5.4
**Region in the US**		
West Coast	4	7.1
Rocky Mountain (all Colorado)	44	78.6

Southwest	2	3.6
South	2	3.6
Mid-Atlantic	4	7.1

Appendix 4. Citizen scientist demographics. For "Region in the US", West Coast included participants from California (N = 2) and Oregon (N = 2); Rocky Mountain participants were all from Colorado (N = 44); Southwest included Nevada (N = 1) and Texas (N = 1); South included participants from Virginia (N = 1) and Kentucky (N = 1); and Mid-Atlantic included participants from New York (N = 2), Delaware (N = 1), and New Jersey (N = 1).
