# Supplementary material for: Bean Cuisine: The Potential of Citizen Science to Help Motivate Changes in Pulse Knowledge and Consumption
Source: Foods. 2023 Jul 11;12(14):2667. doi: 10.3390/foods12142667 (PMC10379374; doi:10.3390/foods12142667)
Supplement: Supplementary file 1 [file foods-12-02667-s001.zip › Supplementary Materials S6 - Updated Bean Cuisine - Nutrient Analysis.pdf]

Menu Template Nutrient Analysis

Total Days: 14
Total Foods: 153

Avg. Daily Kcals: 2037.204
Name: Updated Bean Cuisine

Nutrient	Value	Unit	Goal	%
Weight	2164.500	g		
Kilocalories	2037.204	kcal	2000.000	102 %
Kilojoules	8408.354	kj		
Protein	97.967	g	50.000	196 %
Carbohydrate	292.336	g	275.000	106 %
Available Carbohydrate	0.017	g		
Fat, Total	61.611	g	78.000	79 %
Alcohol	0.277	g		
Cholesterol	208.883	mg	300.000	70 %
Saturated Fat	14.817	g	20.000	74 %
Monounsaturated Fat	19.599	g		
Polyunsaturated Fat	13.948	g		
SFA 4:0	0.220	g		
SFA 6:0	0.153	g		
SFA 8:0	0.096	g		
SFA 10:0	0.225	g		
SFA 12:0, Lauric	0.247	g		
SFA 14:0	0.804	g		
SFA 15:0 Pentadecanoic acid	0.049	g		
SFA 24:0 Lignoceric Acid	0.004	g		
SFA 16:0, Palmitic	6.976	g		
SFA 17:0	0.055	g		
SFA 18:0, Stearic	2.305	g		
SFA 20:0	0.045	g		
SFA 22:0	0.017	g		
MFA 14:1	0.043	g		
MFA 16:1	0.510	g		
MFA 18:1, Oleic	18.300	g		
MFA 20:1	0.170	g		
MFA 22:1	0.011	g		
PFA 18:2, Linoleic	11.574	g		
PFA 18:3, Linolenic	1.769	g		
PFA 18:4	0.004	g		
PFA 20:4	0.108	g		
PFA 20:5, EPA	0.010	g		
PFA 22:5	0.009	g		
PFA 22:6, DHA	0.041	g		
Trans Fatty Acid	0.347	g		
Sum of Trans Fat and Sat Fat	10.419	g		
Omega 3 Polyunsat Fat, Tot	0.000	g		
Omega 6 Polyunsat Fat, Tot	0.000	g		
Sodium	2334.420	mg	2300.000	101 %
Potassium	5291.164	mg	4700.000	113 %
Salt	5.839	g		
Chloride			2300.000	
Vitamin A (RE)	1732.003	RE		

Nutrient	Value	Unit	Goal	%
Cobalamin (Vitamin B12)	5.857	mcg	2.400	244 %
Biotin	20.379	mcg	30.000	68 %
Pantothenic Acid	5.693	mg	5.000	114 %
Vitamin K	290.345	mcg	120.000	242 %
Phosphorus	1543.527	mg	1250.000	123 %
Iodine		mcg	150.000	
Magnesium	495.233	mg	420.000	118 %
Zinc	10.858	mg	11.000	99 %
Copper	1.979	mg	0.900	220 %
Manganese	4.740	mg	2.300	206 %
Selenium	81.964	mcg	55.000	149 %
Fluoride	76.611	mcg		
Chromium	0.051	mg	0.035	145 %
Molybdenum	33.562	mcg	45.000	75 %
Choline	366.456	mg	550.000	67 %
Chlorine		mg		
Sulfur		mg		
Cobalt		mcg		
Boron		mcg		
Nickel		mcg		
Dietary Fiber, Total	61.370	g	28.000	219 %
Soluble Fiber	2.826	g		
Insoluble Fiber	4.471	g		
Crude Fiber	20.556	g		
Sugar, Total	88.964	g		
Added Sugars	11.963	g	50.000	24 %
Glucose	19.675	g		
Galactose	1.264	g		
Fructose	22.073	g		
Sucrose	19.759	g		
Lactose	7.880	g		
Maltose	0.872	g		
Sugar Alcohol	0.000	g		
Other Carbohydrate	0.000	g		
Sorbitol	0.000	g		
Xylitol	0.000	g		
Inositol		mg		
Tryptophan	879.547	mg		
Threonine	2994.376	mg		
Isoleucine	3357.981	mg		
Leucine	5914.066	mg		
Lysine	5212.733	mg		
Methionine	1423.305	mg		
Cystine	883.886	mg		
Phenylalanine	3713.392	mg		
Tyrosine	2417.148	mg		
Valine	4034.569	mg		
Arginine	4643.135	mg		

Nutrient Goal Template
DAILY VALUES/RDI - ADULT/CHILD

Analyzed by
Updated Bean Cuisine

Exchanges

Bread/Starch	2.00
Fat	0.50
Fruit	3.00
Meat-High Fat	0.00
Meat-Medium Fat	0.00
Meat-Very Lean	0.00
Milk-Skim	0.50
Other Carbohydrate	0.00
Vegetables	1.00

Menu Template Nutrient Analysis

Total Days: 14
Total Foods: 153
Avg. Daily Kcals: 2037.204
Name: Updated Bean Cuisine

Nutrient	Value	Unit	Goal	%
Vitamin A (IU)	15483.060	IU	5000.000	310 %
Vitamin A (RAE)	1866.441	mcg	900.000	207 %
Total Carotenoid	0.000	RE		
Beta-Carotene	9282.536	mcg		
Alpha-Carotene	1485.779	mcg		
Lutein (+ Zeaxanthin)	4921.319	mcg		
Beta-Cryptoxanthin	190.685	mcg		
Lycopene	8809.182	mcg		
Vitamin C	199.495	mg	90.000	222 %
Calcium	1494.483	mg	1300.000	115 %
Iron	20.424	mg	18.000	113 %
Vitamin D (ug)	7.463	mcg	20.000	37 %
Vitamin D (IU)	301.091	IU	400.000	75 %
Vitamin E (mg)	0.273	mg	20.000	1 %
Vitamin E (IU)	0.406	IU	30.000	1 %
Vitamin E (Alpha-Tocopherol)	10.528	mg	15.000	70 %
Beta Tocopherol	0.116	mg		
Gamma Tocopherol	3.062	mg		
Delta Tocopherol	0.286	mg		
Alpha Tocotrienol	0.219	mg		
Beta Tocotrienol	0.331	mg		
Gamma Tocotrienol	0.265	mg		
Delta Tocotrienol	0.008	mg		
Thiamin	2.729	mg	1.200	227 %
Riboflavin	2.972	mg	1.300	229 %
Niacin	15.197	mg	20.000	76 %
Niacin Equivalent	32.431	mg	16.000	203 %
Pyridoxine (Vitamin B6)	2.748	mg	1.700	162 %
Folate (Total)	791.023	mcg	400.000	198 %
Folate (DFE)	951.461	mcg	400.000	238 %
Folic Acid	107.096	mcg		
Food Folate	774.177	mcg		

Nutrient	Value	Unit	Goal	%
Histidine	2045.495	mg		
Alanine	3488.060	mg		
Aspartic Acid	8073.089	mg		
Glutamic Acid	13015.740	mg		
Glycine	2948.158	mg		
Proline	4166.004	mg		
Serine	3827.826	mg		
Glutamine	0.000	mg		
Taurine	0.000	mg		
Hydroxyproline	24.793	mg		
Cysteine	0.000	mg		
Moisture	1419.933	g		
Ash	17.377	g		
Caffeine	2.859	mg		
Osmolality		mo		
Theobromine	25.566	mg		
Betaine	24.253	mg		
Phytosterols	50.365	mg		
Stigmasterol	1.308	mg		
Campesterol	2.715	mg		
Beta-sitosterol	22.213	mg		
Epigallocatechin-3-gallate		mg		
Daidzein	0.018	mg		
Genistein	0.029	mg		
Glycitein	0.000	mg		
Total isoflavones	0.053	mg		
Biochanin A	0.090	mg		
Formononetin	0.014	mg		
Coumestrol	0.001	mg		
